# Excessive Drinking Among Men Who Have Sex With Men Recruited From Web-Based Resources: Cross-sectional Questionnaire Study

**DOI:** 10.2196/32888

**Published:** 2022-10-31

**Authors:** César Pérez-Romero, Juan-Miguel Guerras, Juan Hoyos, Marta Donat, Gregorio Barrio, Luis de la Fuente, David Palma, Patricia García de Olalla, María-José Belza, Methysos Project Group

**Affiliations:** 1 Escuela Nacional de Sanidad Instituto de Salud Carlos III Madrid Spain; 2 Centro Nacional de Epidemiología Instituto de Salud Carlos III Madrid Spain; 3 Centro de Investigación Biomédica en Red de Epidemiología y Salud Pública Madrid Spain; 4 Departamento de Salud Pública y Materno-Infantil Universidad Complutense de Madrid Madrid Spain; 5 Servicio de Epidemiología Agència de Salut Pública de Barcelona Barcelona Spain; 6 see Acknowledgments

**Keywords:** alcohol use, men who have sex with men, MSM, dating apps or websites, new recruitment methods, Alcohol Use Disorders Identification Test, AUDIT, hazardous drinking, harmful drinking, binge drinking, alcohol-related problems

## Abstract

**Background:**

US and Northern European studies have found a higher prevalence of alcohol-related problems among men who have sex with men (MSM) than among the general population of men (GPM). However, most of them relied on traditional sampling methods, not profiting from MSM dating apps and websites for recruitment. Besides, analogous comparisons in Southern Europe are lacking.

**Objective:**

This study aimed to compare several indicators of excessive drinking between MSM and GPM in Spain.

**Methods:**

Overall, 5862 MSM were recruited through dating apps or websites for the Méthysos Project, and 10,349 GPM were recruited using probability sampling via the Household Survey on Alcohol and Drugs in Spain from 2018 to 2020. The outcomes were the prevalence of hazardous or harmful drinking (Alcohol Use Disorders Identification Test [AUDIT] ≥8), hazardous drinking (AUDIT-Consumption ≥4), harmful drinking (AUDIT-Problem ≥4), regular hazardous drinking (>14 standard drinks per week), and monthly binge drinking. The prevalence of excessive drinking indicators was calculated for MSM and GPM and compared using the adjusted prevalence ratio (aPR). Two different aPRs and their 95% CIs were estimated using Poisson regression models with robust variance. The first was adjusted for sociodemographic characteristics, and the second was adjusted for the aforementioned covariates plus other drug use.

**Results:**

The prevalence of hazardous or harmful drinking was 15.6% (913/5862) among MSM versus 7.7% (902/10,349) among GPM. After adjusting for sociodemographic covariates, the risk was higher in MSM than in GPM for harmful or hazardous drinking (aPR 1.8, 95% CI 1.6-2.0), harmful drinking (aPR 2.3, 95% CI 2.0-2.7), and binge drinking (aPR 1.7, 95% CI 1.5-1.9); the same in both populations for hazardous drinking (aPR 0.9, 95% CI 0.9-1.0); and higher in GPM than in MSM for regular hazardous drinking (aPR 0.7, 95% CI 0.6-0.9). The relative excess risk of harmful drinking and binge drinking among MSM tended to increase with increasing education level and size of the place of residence, and the opposite was true for the deficit risk in regular hazardous drinking. Additional adjustment for other drug use greatly buffered the relative excess risk in harmful drinking and binge drinking in MSM, while it deepened its deficit risk in regular hazardous drinking.

**Conclusions:**

The use of web-based resources allowed recruiting a large sample of MSM. The risk of hazardous or harmful drinking was 80% greater in MSM than in GPM, which was mainly because of the higher risk of harmful drinking and binge drinking among MSM. Nearly 1 in 6 MSM would benefit from early brief alcohol intervention procedures. The subgroup with harmful or binge drinking combined with other drug use is an important contributor to excess MSM risk in hazardous or harmful drinking and must be a priority target for harm reduction interventions.

## Introduction

In recent years, concerns have been raised about health disparities due to sexual orientation [1-5]. Surveys in the United States have found a higher prevalence of alcohol-related problems among men who have sex with men (MSM) compared with general population of men (GPM) [6-9]. However, studies on the prevalence of excessive drinking and frequency of binge drinking have not found significant increases among MSM [1,2,7,10-13]. Here may be a disparity in alcohol indicators between MSM and GPM depending on what is measured: consumption levels or consequences of alcohol use. It should be noted that there are some exceptions: some studies describe higher consumption levels among certain subgroups of MSM [14-16].

To our knowledge, the only studies in Europe that compared alcohol use between MSM and GPM have been conducted in Northern or Central European countries [2,17-21]. One study in Sweden showed more alcohol-related problems, but not increased consumption, among MSM than among heterosexuals [17]. Another study in Sweden, found a higher prevalence of high-risk alcohol use among homosexuals (men and women jointly) but not among bisexual men [18]. Other studies in the United Kingdom [2,20] or The Netherlands [21] did not observe significant differences in consumption levels, while a recent study in Ireland [22] showed a 3-fold increase in the prevalence of alcohol use disorder in MSM compared with GPM, although 2 different instruments were used.

Knowledge of alcohol use among MSM living in southern European countries is important given that historical cultural norms regarding alcohol use differ between these countries and those where research has been conducted [23]. The Mediterranean consumption pattern, characterized by almost daily drinking (primarily wine accompanying meals), may be less followed by MSM living in Southern European countries compared with GPM, given their higher openness to the cultural habits of other countries. This could affect findings among MSM in terms of both alcohol consumption and related problems.

In contrast, most of the former studies comparing MSM and GPM are based on representative population samples [2,7,9,12,16]. This method, although ensuring probabilistic recruitment, entails as a substantial limitation the small sample of MSM frequently included. We consider that recruitment methods based on new technologies, such as apps and websites, may overcome this problem and increase the size of the MSM sample.

A common problem when comparing drinking indicators between MSM and GPM is the existence of important differences between both groups in sociodemographic characteristics (such as age, place of residence, immigration, or socioeconomic status) that can distort the comparison [24-28]. To avoid this, it is necessary to adjust the comparative measures for these covariates carefully. In addition, the evidence shows that the use of other psychoactive drugs is higher in MSM than in GPM [29], so the difference between comparative measures with and without adjustment for such covariates will allow us to estimate which part of the MSM-GPM differences in drinking indicators can be explained by differences in the use of other drugs.

The Alcohol Use Disorders Identification Test (AUDIT) is a 10-item scale that screens for alcohol use disorders to identify and assist individuals at high risk [30]. It includes several subscales, the most widespread of which is the AUDIT-Consumption (AUDIT-C) (consumption items, Q1-Q3) [31-34]. However, although AUDIT-C performs well as a proxy for hazardous drinking, it is less accurate in identifying alcohol-related problems [35-38], especially within the framework of general population surveys [39]. The complementary subscale AUDIT-Problem (AUDIT-P) (problems items, Q4-Q10) has been shown to screen for these issues as well as full-scale [35]. Some authors have suggested that this two-factor conceptualization (consumption and problems, AUDIT-C and AUDIT-P) is a better fit for scale content than the three-factor structure originally proposed [40].

To the best of our knowledge, only 2 published studies have compared AUDIT scores between MSM and GPM [19,41]. The results were not separated according to consumption or related problems. One of the two studies focused on the full-scale score, whereas the other focused only on AUDIT-C. In contrast, other studies that analyzed AUDIT among MSM did not include a comparative sample of GPM, and none analyzed AUDIT-P separately [42-48].

In this study, we took advantage of dating apps and websites commonly used by MSM to recruit a large sample of MSM and compare various indicators of excessive drinking, including hazardous and harmful drinking, between MSM and GPM in Spain. We used the AUDIT to assess MSM who would potentially benefit from early alcohol interventions. In addition, we aimed to discern the differences between the 2 dimensions of hazardous drinking (distinguishing regular hazardous drinking from binge drinking) and harmful drinking.

## Methods

### Study Design

We conducted a cross-sectional comparison of several measures of excessive drinking between MSM and GPM in Spain. Data were obtained from the results of the AUDIT questionnaire on 2 different samples for MSM and GPM.

### Setting

The MSM sample included participants from the Méthysos Project, which aimed to investigate their health status. Participants were recruited through different web-based resources (see *Participants*, *MSM Sample*) between May and July 2020. The GPM sample was recruited between February and April 2018 within the framework of the Household Survey on Alcohol and Drugs in Spain (Encuesta Sobre Alcohol y Otras Drogas en España [EDADES], 2017 edition) [49].

### Participants

#### MSM Sample

MSM were invited to participate through 3 types of web-based resources:

1. A total of 7 MSM dating apps using promotional banners (Scruff, Grindr), personal messages (GROWLr), or both (Wapo, Bakala, MachoBB, and Xtudr) contributed 70.5% (4655/6602) of the participants.
2. Furthermore, 3 influencers largely followed by the MSM community (Gabriel José Martin–with a video on YouTube available throughout the recruitment period–and @frewaskachannel and @tigrilloig–with 24-hours available stories launched twice (@frewaskachannel) or once (@tigrilloig) during the recruitment period), which contributed 26.3% (1741/6602) of the participants.
3. Finally, a message encouraging the diffusion of the study among friends and acquaintances, placed at the end of the questionnaire, and distribution lists from 3 organizations (Agència de Salut Pública de Barcelona, Pink Peace, and Chem-Safe), which contributed with 3.1% (206/6602) of the participants.

Individuals who decided to participate were addressed to an initial screen where they were informed about the aim and content of the survey. Before starting, they were obliged to check on the “I am of legal age for sexual intercourse and want to participate in the study.” All participants were required to meet the inclusion criteria of being male, at least 16 years old, and having ever had anal intercourse with a male. We provided unique links to each recruitment site to identify the recruitment site for each participant. To avoid multiple participation, the initial screen also included a request to complete the questionnaire only once if the invitation was received in various ways. In addition, to further limit the possibility of multiple responses from one individual, we used the option provided by the software that only allowed the completion of one questionnaire per electronic device. No incentive was offered for participation, limiting the chances of multiple participation. The questionnaire was self-administered, computer- or app-based, and treated for sexual behavior and drug and alcohol use (including AUDIT). The full questionnaire is available in the Multimedia Appendix 1.

In studies using this type of methodology, it is impossible to calculate or estimate the response rate. The apps identify the number of displayed banners and sometimes the number of clicks on the banner, but not the number of different people who see or click on them to obtain some information. Something similar happens with influencers. They may know the number of views on a promotion, but not the number of people, or whether those people meet the criteria to participate. Of the 6602 MSM who began the questionnaire, 740 abandoned it before completing the AUDIT questions; thus, the final sample comprised 5862 MSM.

#### GPM Sample

The GPM sample was obtained from the anonymous database of EDADES, 2017 edition provided by the National Plan on Drugs. The EDADES is a biennial national survey of a representative sample of the population aged 15-64 years living in Spain. It uses a 3-stage random sampling design (census tract, household, and individual sampling). Census tracts and subjects within households were selected using random probabilistic methods. The sample was stratified by age (15-34/35-64) and living region (19 categories). In this edition, people aged 15 to 34 years and living in small regions were oversampled, so analyses were weighted to account for strata imbalance compared with the universe. The global response rate was 50.6%, and the main causes of nonresponse were not opening the door, preventing interviews, and the absence of all household members or the selected person. Before classifying a household or person as absent and selecting another household, the fieldworker should visit the household that was initially selected at least three times on different days and times.

We selected all men aged 16-64 years who participated in the survey (women and men aged 15 years were excluded). EDADES does not have any variables to identify sexual identity or behavior; thus, we could not estimate the proportion of MSM among the group of men participating in the survey, nor excluding them in our analysis. Therefore, we refer to this group as GPM. However, we can infer that this sample is principally composed of heterosexual men, relying on the most recent estimation of the Spanish population on sexual orientation, in which 93.9% (2791/2972) of people declare themselves heterosexual [50]. The EDADES used a self-administered paper-and-pencil questionnaire. The interviewer was present in the household to support the participant if needed. The questions included the AUDIT. We provided the full questionnaire used in the 2017 edition (only in Spanish) in the Multimedia Appendix 2. More information about the survey methodology is available on web [49]. Of the 10,576 GPM who began the questionnaire, 227 (2.1%) abandoned it before completing the AUDIT questions; thus, the final sample comprised 10,349 GPM.

### Variables

Only the participants who had consumed alcohol in the past 12 months answered the AUDIT questionnaire. For the remaining participants, the AUDIT score was imputed as 0. Questionnaire skips were automatically included in the resources used by the MSM sample but had to be manually included in the GPM sample. Textbox 1 presents the AUDIT questionnaire.

This study uses labels and concepts from the recently published 11th revision of the International Classification of Diseases [51] to refer to the different AUDIT measures analyzed. Hazardous alcohol use is defined as “a pattern of alcohol use that appreciably increases the risk of harmful physical or mental health consequences to the user or to others,” while a harmful pattern of use of alcohol is that which “has caused damage to a person’s physical or mental health or has resulted in behavior leading to harm to the health of others.” Even if the AUDIT tool considers the concept of alcohol dependence [30], we opted to include all harm-related questions (dependence and harm) in a single variable for harmful drinking. As explained above, this was done to accomplish the two-domain division of AUDIT (consumption and consequences). The results of the full-scale AUDIT (including questions for hazardous and harmful drinking) were categorized as hazardous or harmful drinking.

Regarding consumption levels, we differentiated a variable of regular hazardous drinking (cumulative weekly exposure to alcohol over a pre-established threshold) from binge drinking to identify differences in drinking patterns. Although weekly alcohol consumption above a certain threshold entails health-related consequences [52], these are expected to be more severe when engaging in binge drinking [53]. Regular hazardous drinking has been named “excessive” or “heavy” drinking elsewhere [15,54], while binge drinking (consumption of more than 5 drinks on one occasion, sometimes equated to 2 hours) is also referred to as “heavy episodic drinking” [12] or “risky single occasion drinking” [25].

**Alcohol Use Disorders Identification Test (AUDIT) questionnaire (English version) [30]. Full-scale AUDIT comprises all questions (score between 0 and 40; utilized threshold: 8), AUDIT-Consumption comprises questions Q1 to Q3 (score 0-12; utilized threshold: 4), and AUDIT-Problem questions Q4 to Q10 (score 0-28; utilized threshold: 4).**

**Q1. How often do you have a drink containing alcohol?**

0. Never (skip to Qs 9-10)

1. Monthly or less

2. 2-4 times a month

3. 2-3 times a week

4. 4 or more times a week

**Q2. How many drinks containing alcohol do you have on a typical day when you are drinking?**

0. 1 or 2

1. 3 or 4

2. 5 or 6

3. 7, 8, or 9

4. 10 or more

**Q3. How often do you have six or more drinks on one occasion?**

0. Never

1. Less than monthly

2. Monthly

3. Weekly

4. Daily or almost daily

Skip to questions 9 and 10 if total score for questions 2 and 3=0

**Q4. How often during the last year have you found that you were not able to stop drinking once you had started?**

0. Never

1. Less than monthly

2. Monthly

3. Weekly

4. Daily or almost daily

**Q5. How often during the last year have you failed to do what was normally expected from you because of drinking?**

0. Never

1. Less than monthly

2. Monthly

3. Weekly

4. Daily or almost daily

**Q6. How often during the last year have you needed a first drink in the morning to get yourself going after a heavy drinking session?**

0. Never

1. Less than monthly

2. Monthly

3. Weekly

4. Daily or almost daily

**Q7. How often during the last year have you had a feeling of guilt or remorse after drinking?**

0. Never

1. Less than monthly

2. Monthly

3. Weekly

4. Daily or almost daily

**Q8. How often during the last year have you been unable to remember what happened the night before because you had been drinking?**

0. Never

1. Less than monthly

2. Monthly

3. Weekly

4. Daily or almost daily

**Q9. Have you or someone else been injured because of your drinking?**

0. No

2. Yes, but not in the last year

4. Yes, during the last year.

**Q10. Has a relative or friend or a doctor or another health worker been concerned about your drinking or suggested you cut down?**

0. No

2. Yes, but not in the last year

4. Yes, during the last year.

### Quantitative Variables

Scores for the full-scale AUDIT (hazardous or harmful drinking), AUDIT-C (hazardous drinking), and AUDIT-P (harmful drinking or alcohol-related problems) instruments were calculated by adding the scores in Q1-10, Q1-Q3, and Q4-Q10, respectively. The weekly average number of standard drinks was used as an indicator of the intensity of regular alcohol consumption. It was estimated by multiplying the answers for questions on drinking frequency (Q1) and quantity (Q2). To this end, the median values of each response category of Q1 and Q2 were used, except for the extreme categories “monthly or less” in Q1, where 0.25 drinking days per week was assigned, and “ten or more were assigned to Q2, where 10 standard drinks per day were assigned. A similar method was used previously [11]. The occurrence of binge drinking at least monthly was obtained from the AUDIT Q3 on binge drinking frequency. This method has been used elsewhere [55,56].

### Statistical Methods

For the GPM sample, to account for strata imbalance compared with the universe, data were weighted by region (19 categories), size of place of residence (7 categories), age (7 categories), and sex (2 categories), resulting in 619 different values for the ponderation factor (from 0.04 to 3.06). For the MSM sample, as the global universe of MSM residing in Spain was unknown, all participants were assigned a ponderation factor of 1. An initial descriptive analysis of the general characteristics of the MSM and GPM samples was performed, and the statistical significance of the differences was assessed using the Pearson *χ^2^* test. The main outcomes were the prevalence of harmful or hazardous drinking (AUDIT score≥8), hazardous drinking (AUDIT-C score≥4), harmful drinking or alcohol-related problems (AUDIT-P score≥4), regular hazardous drinking (>14 standard drinks per week or >20 g of pure alcohol per day in a year), and monthly binge drinking (occurrence of binge drinking at least monthly in a year). The AUDIT cutoff was originally established by the World Health Organization [24], and the AUDIT-C has been widely used [27,42]. As there are no well-established cutoffs for AUDIT-P, we adapted the cutoff used by Hansen et al [52] considering the recommendations of the lower thresholds [24,29]. We selected the threshold for regular hazardous drinking based on updated recommendations from national and international guidelines regarding limits on low-risk alcohol consumption [53-55].

The prevalence of the different drinking indicators was calculated in both MSM and GPM and compared between the groups using the adjusted prevalence ratio (aPR). aPR is a measure of relative inequality without units, which indicates the number of times the risk is higher or lower in MSM compared with GPM. Two different aPRs and their corresponding 95% CI were estimated using Poisson regression models with robust variance [57]. The first was adjusted for age, education level, size of place of residence, country of birth, and perceived economic status (Model I), and the second for the aforementioned covariates plus the use of other drugs in the past 12 months (Model II). Analyses were carried out globally and by age group (16-24, 25-34, and 35-64 years), education level (university or no university), and size of their place of residence (<10,000, 10,000-500,000, and >500,000 inhabitants). Categories were chosen following usual classifications and to obtain a differentiated profile in young men (16-24 years) versus other groups, or in rural areas (<10,000 inhabitants), and large cities (>500,000 inhabitants) as opposed to other living conditions. Differences and comparative measures were considered statistically significant if 2-tailed *P*-values were <.05. The analysis was not preregistered, and the results should be considered exploratory. Statistical analyses were performed using Stata version 15 (StataCorp).

### Ethical Considerations

This study was approved by the Research Ethics Committee of the Instituto de Salud Carlos III (Comité de Ética de la Investigación [research ethics committee] Proyecto de Investigación [research project; principal investigator] 35_2020-v3). Concerning the MSM sample, individuals approved their conformity to participate by clicking on “On my age, I am legally authorized to have sexual relations and I want to participate in the study.” This message appeared before starting the questionnaire, as can be observed in the (Multimedia Appendix 3). Privacy was ensured by not asking for personal data, which led to possible identification. Regarding the GPM sample, individuals selected by randomized sampling had the possibility of denying participation. For those who wanted to participate, the interviewers provided informed consent before starting the study.

## Results

### Baseline Characteristics

Participants were more frequently aged 25-34 years (MSM) and 35-44 years (GPM). Compared with the GPM, they had a higher education level, lived more frequently in urban settings, and declared a better economic status. The prevalence of alcohol use in the past 12 months was similar between MSM and GPM, but that of other drugs was much higher among MSM (Table 1).

**Table 1:** Baseline characteristics of the sample: men who have sex with men (MSM) and general population men (GPM), Spain, 2018-2020.

Characteristics		MSM (N=5862), n (%^a^)	GPM (N=10,349), n (%^a,b^)	*P* value
**Age group (years)**				<.001
	16-24	821 (14.0)	1955 (18.9)	
	25-34	1650 (28.1)	2701 (26.1)	
	35-44	1625 (27.7)	2665 (25.8)	
	45-54	1265 (21.6)	1668 (16.1)	
	55-64	501 (8.5)	1360 (13.1)	
**Education level**				.001
	≤Lower secondary	586 (10.0)	5109 (49.6)	
	Upper secondary	2104 (35.9)	3424 (33.2)	
	University	3169 (54.1)	1767 (17.2)	
**Place of birth**				<.001
	Spain	4827 (82.3)	9116 (93.0)	
	Latin America	797 (13.6)	110 (1.1)	
	Other	238 (4.1)	580 (5.9)	
**Size of place of residence (inhabitants)**				<.001
	>1 million	1689 (31.6)	638 (6.2)	
	500,000-1 million	617 (11.5)	770 (7.4)	
	100,000-500,000	1155 (21.6)	2142 (20.7)	
	50,000-100,000	528 (9.9)	1613 (15.6)	
	10,000-50,000	734 (13.7)	2758 (26.6)	
	<10,000	622 (11.6)	2428 (23.5)	
**Perceived economic status**				<.001
	Good	3447 (64.4)	3379 (48.3)	
	Regular	1462 (27.3)	2732 (39.0)	
	Bad	443 (8.3)	886 (12.7)	
Alcohol use in past 12 months		4729 (80.7)	8129 (78.5)	.046
Other drug use in past 12 months		3529 (72.6)	2402 (23.6)	<.001

^a^Percentages were estimated over cases with valid data.

^b^Data were weighted by region, size of place of residence, age, and sex.

### Hazardous Drinking and Harmful Drinking

The prevalence of hazardous or harmful drinking among MSM was 15.6% (913/5862) compared with 7.7% (902/10,349) in the GPM group, leading to an aPR of 1.8 (95% CI 1.6-2.0). Significant MSM-GPM disparities in prevalence in the same direction were observed in all subgroups, except for those who did not consume other drugs in the past 12 months (Table 2). The prevalence of hazardous drinking was similar for MSM (1950/5862, 33.3%) and GPM (3472/10,349, 34.1%; aPR 0.9, 95% CI 0.9-1.0). A slight disparity favoring GPM was observed in several subgroups: those aged 35 to 64, with no university education level, and living in places with <10,000 inhabitants. Moreover, both MSM users and nonusers of other drugs were less frequent hazardous drinkers than their GPM counterparts (Table 2).

**Table 2:** Comparison of the prevalence of 3 Alcohol Use Disorders Identification Test (AUDIT) measures between men who have sex with men (MSM) and general population men (GPM), by age, education level, size of place of residence and other drug use, Spain, 2018-2020^a^.

						MSM, n (%)			Sample size (n)			GPM, n (%^a^)			Sample size (n)			aPR^b^ (95% CI)^c^
**Hazardous or harmful drinking (AUDIT score ≥8)**																		
	**All participants**				913 (15.6)			5862			902 (7.7)			10,349			1.8 (1.6-2.0)	
		**Age (years)**																
			16-24	184 (22.4)			821			216 (10.4)			1955			1.5 (1.2-2.0)		
			25-34	310 (18.8)			1650			275 (9.4)			2701			1.8 (1.5-2.3)		
			35-64	419 (12.4)			3391			411 (6.7)			5693			1.8 (1.5-2.2)		
		**Education level**																
			No university	437 (16.2)			2690			773 (8.1)			8533			1.8 (1.5-2.1)		
			University	475 (15.0)			3169			125 (6.0)			1767			1.7 (1.3-2.3)		
		**Size of place of residence (inhabitants)**																
			<10,000	73 (11.7)			622			213 (7.9)			2428			1.5 (1.1-2.0)		
			10,000-500,000	338 (14.0)			2417			566 (7.7)			6513			1.8 (1.5-2.1)		
			>500,000	385 (16.7)			2306			123 (7.5)			1408			2.2 (1.7-2.9)		
		**Other drug use in past 12 months**																
			No	67 (5.0)			1332			443 (5.0)			7797			0.9 (0.7-1.3)		
			Yes	755 (21.4)			3529			450 (16.9)			2402			1.2 (1.0-1.5)^d^		
**Hazardous drinking (AUDIT-Consumption score ≥4)**																		
	**All participants**				1950 (33.3)			5862			3472 (34.1)			10,349			0.9 (0.9-1.0)	
		**Age**																
			16-24	316 (38.5)			821			595 (30.2)			1955			1.1 (0.9-1.3)		
			25-34	633 (38.4)			1650			923 (34.0)			2701			1.1 (1.0-1.3)		
			35-64	1001 (29.5)			3391			1954 (35.0)			5693			0.9 (0.8-0.9)		
		**Education level**																
			No university	849 (31.6)			2690			2900 (34.9)			8533			0.9 (0.8-1.0)^d^		
			University	1100 (34.7)			3169			554 (30.4)			1767			1.0 (0.9-1.1)		
		**Size of place of residence (inhabitants)**																
			<10,000	188 (30.2)			622			946 (39.2)			2428			0.8 (0.7-0.9)		
			10,000-500,000	764 (31.6)			2417			2064 (32.9)			6513			1.0 (0.9-1.0)		
			>500,000	815 (35.3)			2306			462 (32.4)			1408			1.0 (0.9-1.2)		
		**Other drug use in past 12 months**																
			No	219 (16.4)			1332			2303 (30.5)			7797			0.6 (0.5-0.7)		
			Yes	1451 (41.1)			3529			1121 (46.3)			2402			0.9 (0-8-0.9)		
**Harmful drinking (AUDIT-Problem score ≥4)**																		
	**All participants**				715 (12.2)			5862			604 (5.0)			10,349			2.3 (2.0-2.7)	
		**Age (years)**																
			16-24	173 (21.1)			821			164 (7.6)			1955			2.3 (1.7-3.1)		
			25-34	231 (14.0)			1650			177 (6.1)			2701			2.1 (1.6-2.7)		
			35-64	311 (9.2)			3391			263 (4.2)			5693			2.4 (1.9-3.0)		
		**Education level**																
			No university	366 (13.6)			2690			527 (5.4)			8533			2.2 (1.8-2.6)		
			University	348 (11.0)			3169			74 (3.6)			1767			2.6 (1.7-3.8)		
		**Size of place of residence (inhabitants)**																
			<10,000	60 (9.6)			622			129 (5.0)			2428			1.8 (1.2-2.6)		
			10,000-500,000	270 (11.2)			2417			403 (5.2)			6513			2.1 (1.7-2.6)		
			>500,000	296 (12.8)			2306			72 (4.3)			1408			3.4 (2.4-4.7)		
		**Other drug use in past 12 months**																
			No	61 (4.6)			1332			260 (2.9)			7797			1.4 (1.0-2.0)^d^		
			Yes	583 (16.5)			3529			336 (12.3)			2402			1.5 (1.2-1.8)		

^a^Crude prevalence. For the GPM sample, data were weighted by region, size of place of residence, age, and sex.

^b^aPR: adjusted prevalence ratio.

^c^aPR were obtained from Poisson regression with robust variance in the framework of generalized linear models and adjusted by age, education level, size of place of residence, country of birth and economic status. The reference group (aPR=1) was general population men.

^d^*P*<.05.

### Regular Hazardous Drinking and Binge Drinking

The prevalence of regular hazardous drinking (>14 drinks per week) was lower among MSM (245/5862, 4.2%) than among GPM (524/10,349, 5.8%), with an aPR of 0.7 (95% CI 0.6-0.9). A statistically significant MSM-GPM disparity in such prevalence was not observed among individuals aged between 16 and 34 years, with university education level, or living in places with >500,000 inhabitants (Table 3).

The prevalence of monthly binge drinking was higher among MSM (798/5862, 13.6%) than among GPM (855/10,349, 7.6%), with an aPR of 1.7 (95% CI 1.5-1.9). Significant MSM-GPM disparities in prevalence in the same direction were observed in all subgroups, except for other drug users (Table 3).

Finally, the prevalence of harmful drinking was higher among MSM (715/5862, 12.2%) than among GPM (604/10,349, 5.0%), with an aPR of 2.3 (95% CI 2.0-2.7) Significant MSM-GPM disparities in such prevalence in the same direction were observed in all subgroups (Table 2).

The aPRs of Model II after further adjustment for the use of other drugs in the past 12 months are shown in Tables S1 and S2 in Multimedia Appendix 4. The considerable relative excess prevalence in MSM found in Model I for both harmful and binge drinking was greatly dampened in Model II. Thus, the aPR went from 2.3 (95% CI 2.0-2.7) in Model I to 1.5 (95% CI 1.3-1.7) in Model II for harmful drinking, and from 1.7 (95% CI 1.5-1.9) to 1.2 (95% CI 1.0-1.3) for binge drinking. Regarding regular hazardous drinking, the relative prevalence deficit in MSM found in Model I increased in Model II. Thus, aPR went from 0.7 (95% CI 0.6-0.9) in Model I to 0.5 (95% CI 0.4-0.6) in Model II. In contrast, the aPR for hazardous drinking went from 0.9 (95% CI 0.9-1.0) in Model I to 0.8 (95% CI 0.7-0.8) in Model II, and that of hazardous or harmful drinking, from 1.8 (95% CI 1.6-2.0) to 1.2 (95% CI 1.0-1.4).

**Table 3:** Comparison of the prevalence of regular hazardous drinking and monthly binge drinking between men who have sex with men (MSM) and general population men (GPM), by age, education level, size of place of residence, and other drug use, Spain, 2018-2020^a^.

							MSM, n/N (%)		GPM, n/N (%)		aPR^b^ (95% CI)^c^
**Regular hazardous drinking (>14 drinks per week)**											
	**All participants**					245/5862 (4.2)		524/10,349 (5.8)		0.7 (0.6-0.9)	
		**Age (years)**									
					16-24	25/821 (3.1)		43/1955 (2.2)		0.6 (0.3-1.2)	
					25-34	75/1650 (4.5)		103/2701 (4.2)		0.9 (0.6-1.4)	
					35-64	145/3391 (4.3)		378/5693 (7.0)		0.6 (0.5-0.8)	
		**Education level**									
					No university	104/2690 (3.9)		470/8533 (6.4)		0.6 (0.5-0.8)	
					University	141/3169 (4.4)		53/1767 (3.5)		0.8 (0.5-1.2)	
		**Size of place of residence (inhabitants)**									
					<10,000	13/622 (2.1)		173/2428 (7.2)		0.3 (0.2-0.6)	
					10,000-500,000	77/2417 (3.2)		286/6513 (5.6)		0.6 (0.5-0.9)	
					>500,000	106/2306 (4.6)		65/1408 (4.8)		1.1 (0.7-1.7)	
		**Other drug use in past 12 months**									
					No	13/1332 (1.0)		297/7797 (4.4)		0.3 (0.2-0.6)	
					Yes	212/3529 (6.0)		221/2402 (10.6)		0.5 (0.4-0.6)	
**Binge drinking (at least monthly)**											
	**All participants**					798/5862 (13.6)		855/10,349 (7.6)		1.7 (1.5-1.9)	
		**Age (years)**									
			16-24			144/821 (17.5)		200/1955 (10.2)		1.7 (1.2-2.3)	
			25-34			270/1650 (16.4)		266/2701 (9.3)		1.7 (1.3-2.1)	
			35-64			384/3391 (11.3)		389/5693 (6.7)		1.6 (1.3-2.0)	
		**Education level**									
			No university			372/2690 (3.8)		740/8533 (8.1)		1.6 (1.4-1.9)	
			University			426/3169 (13.4)		111/1767 (5.5)		1.8 (1.3-2.4)	
		**Size of the place of residence (inhabitants)**									
				<10,000		70/622 (11.3)		218/2428 (8.1)		1.4 (1.0-1.9)^d^	
				10,000-500,000		307/2417 (12.7)		527/6513 (7.5)		1.7 (1.4-2.1)	
				>500,000		314/2306 (13.6)		110/1408 (7.6)		1.8 (1.4-2.4)	
		**Other drug use in the past 12 months**									
			No			70/1332 (5.3)		453/7797 (5.4)		1.0 (0.7-1.3)	
			Yes			645/3529 (18.3)		393/2402 (15.4)		1.2 (1.0-1.4)	

^a^Crude prevalence. For the GPM sample, data were weighted by region, place of residence, age, and sex.

^b^aPR: adjusted prevalence ratio.

^c^aPR was obtained from Poisson regression with robust variance in the framework of generalized linear models and adjusted by age, education level, size of place of residence, country of birth, and economic status. The reference group (aPR=1) was general population men.

^d^*P*<.05.

## Discussion

### Principal Findings

Compared with GPM, we found a relative excess risk of hazardous or harmful drinking (full-scale AUDIT) among MSM living in Spain. Nearly 1 in 6 MSM had hazardous or harmful drinking and would benefit from early brief alcohol intervention procedures (the ultimate goal for which the AUDIT tool was designed) [30]. The MSM’s excess risk was mainly because of harmful drinking and binge drinking. In contrast, both populations had similar risks for hazardous drinking (AUDIT-C), resulting from the balance of a higher risk of regular hazardous drinking among GPM than MSM, and the opposite for binge drinking.

The relative excess risk in the GPM of hazardous drinking and regular hazardous drinking tended to increase among older, less educated, and rural-dwelling individuals. In contrast, the relative excess risk of harmful and binge drinking in MSM tended to be larger among individuals aged 16-24 years (only in the case of harmful drinking) or with university education level and living in big cities (in both cases).

Additional adjustment for other drug use greatly buffered the considerable relative excess risk in MSM compared to that in GPM for harmful drinking and binge drinking while deepening the risk deficit in MSM for regular hazardous drinking. This suggests that drinking behaviors and other drug use were strongly associated in the studied populations. Therefore, the considerable excess of risk in MSM compared with GPM for binge drinking and harmful drinking is mainly because of the subgroup having both these drinking patterns and the use of other drugs. This subgroup represents a much higher proportion of the total in MSM than in GPM, and must clearly be a priority target for harm reduction interventions.

### Comparison With Previous Work

This is the first study to directly compare excessive drinking indicators (hazardous and harmful drinking) between MSM and GPM in a southern European country. Moreover, this is the first study ever published that differentiates between these 2 dimensions using a validated instrument (AUDIT). Moreover, because of the use of new technologies in the recruitment process, namely apps and websites, it includes a larger sample of MSM than that in most previous studies inside and outside Europe [1,2,6,7,9-18,41,58].

Only one study has compared hazardous or harmful drinking between these 2 populations using full-scale AUDIT [19], and the results showed a lower mean AUDIT score among MSM than among heterosexual men. In this study, both populations were recruited using snowball sampling and the study did not include a stratified analysis to distinguish between the 2 dimensions evaluated here. Other studies [42-48] have estimated the proportion of MSM who engage in hazardous or harmful drinking using AUDIT but did not compare it to that of GPM. The proportion of hazardous or harmful drinking (full-scale AUDIT) in nearly all these studies was higher than that in our study, and it is likely because of the fact that these studies included individuals with a higher risk of unhealthy behaviors. However, we believe that it does not make much sense to compare these prevalences, nor those of the 2 dimensions separately, because the levels and patterns of alcohol consumption are primarily determined by the social environment in which one lives. From our perspective, the most relevant measures to use are relative comparisons of MSM and their heterosexual counterparts, such as aPR.

There are 3 studies [7,12,17] that have used instruments or indicators other than AUDIT, which estimated both dimensions separately. Their results showed low or insignificant differences in hazardous drinking but higher and significant differences in harmful drinking among MSM compared with GPM. This study also found the same clear disparity between the two dimensions.

One study [41] compared only hazardous drinking using AUDIT-C and found a higher but not statistically significant prevalence in MSM compared with heterosexual men, which agrees with the results of various studies using other indicators [1,10,11]. However, the MSM sample size used in these studies tends to be limited, as is usually observed in studies derived from general population surveys. It was not possible to compare our results in terms of regular alcohol consumption with those of 2 other studies that also performed this analysis because their results were presented [2,15]. Our findings suggest that higher binge drinking among MSM contradicts the findings of most prior studies, which have reported no such differences between MSM and GPM [1,2,7,13], or an even lower prevalence among certain subgroups of MSM [10,12].

No other study has analyzed harmful drinking using AUDIT-P; however, our findings of a high aPR MSM or GPM are similar to those previously described in studies using Diagnostic and Statistical Manual of Mental Disorders criteria [6-8].

Considering these findings, we can affirm that there is a slight difference in hazardous drinking between MSM and GPM, but a remarkable increase in harmful drinking among MSM also exists in Mediterranean countries such as Spain. However, the reasons for this discrepancy are not well understood. In the case of Spain, it could be that MSM adopted Northern European drinking patterns more quickly, characterized by a higher frequency of binge drinking, which is more likely to produce harmful consequences [53]. As an indicator of more traditional habits, in our sample, the Mediterranean alcohol consumption pattern was predominant among older, lower-educated, and rural-dwelling GPM. The changes in consumption patterns among MSM may be related to their greater knowledge of habits in other countries because of their openness to cultural differences and the higher proportion of young, university-educated, large city dwellers in this group. Attributing differences between homosexuals and heterosexuals to differential consumption patterns was mentioned in one of the first studies on this issue, whose author suggested a higher frequency of “European and continental drinking patterns” among Swedish MSM [17]. Nevertheless, there were no differences in consumption patterns between MSM and GPM.

In contrast, minority stress theory has also been proposed to explain the increase in harmful and hazardous drinking among MSM [59,60]. According to this theory, alcohol and other drug use is a response to psychological discomfort derived from stigma or internalized homophobia among lesbian, gay, bisexual, transgender community [61]. As our study did not explore psychological variables or drinking motives, further studies are needed to interpret our findings in terms of this theory.

Other factors may have influenced the results. It is well known that certain potentially alcohol-linked phenomena, such as sex work [62], chemsex [63], or sex tourism [64] are more frequent among MSM. Even though only 13.0% (431/3319) of MSM in our sample (data not shown, nonrespondents excluded from the denominator) declared engaging regularly in binge drinking during chemsex sessions, and only 15.3% (898/5854) affirmed having received economic rewards for sex more than once in their life course, the influence of these variables warrants further investigation. Concerning sex tourism, 92.8% (596/642) of foreign MSM in our sample declared living in Spain for at least 1 year, so their consideration as tourists can be ruled out. Moreover, all analyses were adjusted for country of birth.

In summary, it could be that there are differences in consumption patterns between MSM and GPM that have not been properly analyzed, or, more probably, that MSM are more susceptible to the harmful effects of alcohol use or that they have a greater ability to identify or disclose them. Further research is needed to clarify this last point, which requires the use of large samples for both comparison groups. Studies based on general population surveys, which represent the majority to date, do not permit discerning of these particular issues and often include a small number of MSM.

Finally, although not an objective of this study, our findings suggest that AUDIT measures 2 different domains [40]. It also illustrates the inadequacy of using the AUDIT-C scale in isolation to assess differences in alcohol-related problems among MSM [35].

### Limitations

The use of 2 different sampling methods for MSM and GPM is a limitation of this study. However, using dating apps and websites for the recruitment of MSM was a way to overcome 2 handicaps: the small sample of MSM frequently included in studies based on representative samples of the general population and the fact that the national drug survey in Spain does not include questions about sexual orientation. Although this sampling method may have overrepresented hazardous drinkers among MSM, it provided a significantly larger MSM sample than that in most studies with probabilistic sampling methods, in which the inclusion of a large number of MSM was hindered by the low percentage of men who reported having sex with other men. In our study, the MSM questionnaire was self-administered, which is not always guaranteed in general population surveys. This might have reduced the probability of reporting bias for nonnormative behaviors, such as homosexual behavior or alcohol use.

Another limitation related to recruitment is the different contexts in which alcohol was more frequently consumed in these 2 groups. Even though dating apps as well as other Internet-based resources were used to recruit MSM, a skew toward “party-goers” may have taken place. As some questions in AUDIT (notably in AUDIT-P, such as Q6 or Q8) are related to nightlife drinking, this point may have had an influence. Unfortunately, we did not have a variable to measure the context of consumption. On the contrary, we do not think that recruiting MSM, but not GPM, during the COVID-19 pandemic (between May and July 2020) may have increased the differences favoring MSM. It has been observed that hazardous drinking increased during lockdowns [65-68], but AUDIT questions refer to common habits in the last 12 months, so most times evaluated had occurred out of the general lockdown in March and April 2020.

Another limitation is related to AUDIT thresholds. Cutoffs are needed for screening; however, when different cutoff points are used, epidemiological research is hindered. We offset this limitation by estimating new results by applying different cutoffs used in other studies. In addition, problems related to thresholds were not exclusive to AUDIT; they also appeared when establishing the recommended levels of weekly alcohol consumption. The final limitation relates to the use of only 2 AUDIT questions (quantity and frequency) to estimate alcohol consumption. Although this was not the first study to measure this variable using these 2 questions (or similar questions) [11], a more complete module concerning consumption in the 2 surveys would have provided more detailed results.

### Conclusions and Implications for Intervention and Research

This is the first study to compare indicators of hazardous and harmful drinking (problems or consequences) between MSM and GPM living in southern Europe. The use of different Internet-based resources in recruitment (mainly apps and influencers) allowed us to obtain information on alcohol use from a large and diverse sample of MSM. Using a validated brief screening tool (AUDIT), we found an 80% excess risk of hazardous or harmful drinking among MSM, which was mainly owing to the higher risk of harmful drinking and binge drinking among MSM.

From the perspective of the implications for clinical practice, it is important to emphasize that the periodic use of brief screening tools such as AUDIT would seem to be highly recommended in health consultations of MSM. This would result in a significant proportion of MSM benefiting from early brief alcohol harm reduction interventions and from referral to specialized services when needed. Harm reduction strategies should focus on binge drinking. Nursing professionals in primary care and sexual transmitted infections or HIV clinics (which take care of a large number of MSM) may provide appropriate services for the implementation of these screening instruments.

From the perspective of future research, it is desirable to enrich these findings by conducting studies based on a single population-based survey. To achieve this goal, surveys in Spain (and in other countries) should include a variable on sexual behavior or gender identity, as it has been common in the United States for a decade. Second, we want to highlight that there are currently no clear explanations for the disparity in the findings in the 2 domains of AUDIT. Neither the tendency toward incorporation of non-Mediterranean consumption patterns among MSM nor the minority stress theory satisfactorily explains these results. Therefore, further studies are needed to analyze whether differential consumption patterns exist or whether there are different levels of susceptibility to alcohol use and its harmful consequences.

## Appendix

Multimedia Appendix 1 Men who have sex with men sample questionnaire.

Multimedia Appendix 2 General population of men sample questionnaire Encuesta Sobre Alcohol y Otras Drogas en España (Spanish National Survey on Alcohol and Drugs).

Multimedia Appendix 3 Message appeared before starting the questionnaire Méthysos.

Multimedia Appendix 4 Supplementary tables.

## Abbreviations

aPR: adjusted prevalence ratio
AUDIT: Alcohol Use Disorders Identification Test
AUDIT-C: Alcohol Use Disorders Identification Test-Consumption
AUDIT-P: Alcohol Use Disorders Identification Test-Problem
EDADES: Encuesta Sobre Alcohol y Otras Drogas en España
GPM: general population of men
MSM: men who have sex with men
