# Supplementary material for: Excessive Drinking Among Men Who Have Sex With Men Recruited From Web-Based Resources: Cross-sectional Questionnaire Study
Source: JMIR Public Health Surveill. 2022 Oct 31;8(10):e32888. doi: 10.2196/32888 (PMC9664322; doi:10.2196/32888)
Supplement: Multimedia Appendix 1 [file publichealth_v8i10e32888_app1.zip › MSM sample questionnaire_Computer_Spanish.pdf]

Pantalla 1a de presentación:

Somos investigadores del Instituto de Salud Carlos III. Estamos realizando un estudio financiado por el Ministerio de Sanidad, dirigido a hombres que han tenido sexo con otros hombres. Nuestro objetivo es conocer las características del consumo de distintos tipos de sustancias con fines recreativos o para sexo e identificar riesgos y consecuencias negativas para la salud, así como medidas de prevención y uso de servicios de salud El estudio ha sido aprobado por el comité de ética de nuestra institución.

Sólo tendrías que contestar a una encuesta completamente **ANÓNIMA**: No recogemos direcciones IP ni información que permita tu identificación.

Contéstala una única vez, aunque la invitación te llegue por varios sitios.

Te llevará alrededor de 10-15 minutos.

Pantalla 1b de presentación:

Se contesta mucho más rápido y cómodo en **tablet u ordenador**. Por eso:

- Si nos estás leyendo en **tablet u ordenador** pulsa en este link para comenzar: https://es.surveymonkey.com/r/online_v4
- Si estás con un **móvil**:
  - Para contestarla en tablet u ordenador, copia el link anterior y enviátelo a tu a tu correo o whatsapp (te lo recomendamos)
  - Si prefieres contestar ahora con el móvil, pincha aquí: https://es.surveymonkey.com/r/onlinemovil_v3

Pantalla 2 de entrada al cuestionario: Para ordenador o tablet

Para participar por favor, marca la siguiente casilla

☐ Tengo la edad legal para tener relaciones sexuales y quiero participar en el estudio.

Antes de empezar **RECUERDA**:

- Para moverte por la encuesta usa siempre los botones de ANTERIOR y SIGUIENTE que tienes después de cada pregunta.
- **NO** emplees el botón de retroceso del navegador, te puede sacar de la encuesta y hay que volver a empezar
- Nunca dejes una pregunta en blanco porque dudes, selecciona la que te parezca más adecuada, aunque no estés del todo seguro.

Eres... Q1. [Respuesta obligatoria]

1. Hombre
2. Mujer
3. Trans

[Si Q1=2 OR 3] **Q1b. Esta investigación está dirigida a hombres. Estás invitado/a a leer y completar el resto de la encuesta, sin embargo, no podremos utilizar tus datos. ¿Qué te gustaría hacer?**

1. Salir de la encuesta [Salir de la encuesta]
2. Seguir leyendo

[If Q1b=1] Muchas ***Gracias por tu interés en el proyecto***

¿En qué país naciste?

1. España
2. Otro----- Especificar: _________________

¿Qué edad tienes? [Respuesta obligatoria]

/__/__/ Si tiene menos de 16 años → No tienes la edad legal suficiente para tener relaciones sexuales y por lo tanto no puedes participar en el estudio.

¿En qué país vives actualmente?

3. España
4. Otro----- Especificar: _________________[salta 1 pregunta]

¿Y en qué provincia? **Desplegable de provincias**

¿Cuál es el máximo nivel de estudios que has completado?

1. Ninguno o educación primaria (el nivel que debe acabarse a los 12 años)
2. Educación Secundaria Obligatoria de primer y segundo ciclo. (el nivel que debe acabarse a los 16 años)
3. Enseñanza secundaria superior; Bachillerato, Ciclo Formativo de Grado Medio. (el nivel que debe acabarse a los 18 años)
4. Ciclo Formativo de Grado Superior o equivalente (el nivel que debe acabarse a los 20 años)
5. Enseñanza Universitaria. Diplomatura, Licenciatura, Grado, Máster, Doctorado

SOBRE SEXUALIDAD

El término "relaciones sexuales "o "tener sexo" puede aplicarse a conceptos diversos.

Aquí consideramos SÓLO cuando existe penetración anal (activa o pasiva) o vaginal.

Teniendo en cuenta la definición anterior, a lo largo de tu vida, ¿has tenido relaciones sexuales? [Respuesta obligatoria]

1. No nunca, ni con hombres ni con mujeres [Salir de la encuesta]
2. Sólo con mujeres [Salir de la encuesta]
3. Más a menudo con mujeres, pero al menos una vez con un hombre [salta 1 pregunta] *(filtro mujeres)*
4. Igual con hombres que con mujeres [salta 1 pregunta] *(filtro mujeres)*
5. Más a menudo con hombres, pero al menos una vez con una mujer [salta 1 pregunta] *(filtro mujeres)*
6. Solo con hombres [salta 1 pregunta] *(Filtro de ocultar las preguntas de mujeres)*

Este cuestionario está destinado EXCLUSIVAMENTE a hombres que HAN TENIDO sexo con hombres y SÓLO si ha incluido penetración anal.

Estás invitado a leer y completar el resto de la encuesta, sin embargo, no podremos utilizar tus datos. ¿Qué te gustaría hacer?

1. Salir de la encuesta [Salir de la encuesta]
2. Seguir leyendo

De las siguientes opciones, ¿Cuál describe mejor la forma en la que vives tu vida sexual con los hombres?

1. Abiertamente
2. Discretamente
3. Escondida
4. En secreto total

Recuerda "relaciones sexuales" o "tener sexo": SÓLO hay penetración anal o vaginal.

¿A qué edad tuviste tu PRIMERA relación sexual con un hombre? /__/__/

EN LOS ÚLTIMOS 12 MESES ¿con cuántos hombres has tenido relaciones sexuales sin preservativo?

1. Ninguno
2. Uno
3. Menos de 5
4. Menos de 10
5. Menos de 20
6. Menos de 50
7. Menos de 100
8. Menos de 200
9. Más de 200

En tu vida, ¿cuántas veces has pagado a un hombre para tener sexo contigo? Por pagar entendemos que diste dinero, regalos o le hiciste favores a cambio de sexo.

1. Ninguna *[salta 1 pregunta]*
2. Una
3. Menos de 5
4. Menos de 10
5. Menos de 20
6. Menos de 50
7. Menos de 100
8. Más de 100

¿Cuándo fue la última vez?

1. En el último mes
2. En los últimos 6 meses
3. En los últimos 12 meses
4. En los últimos 5 años
5. Hace más de 5 años

En tu vida, ¿cuántas veces te ha pagado un hombre para tener sexo contigo?

1. Ninguna *[salta 2 preguntas]*
2. Una
3. Menos de 5

4. Menos de 10
5. Menos de 20
6. Menos de 50
7. Menos de 100
8. Más de 100

¿Qué edad tenías la primera vez que lo hiciste?

/__/__/

¿Cuándo fue la última vez?

1. En el último mes
2. En los últimos 6 meses
3. En los últimos 12 meses
4. En los últimos 5 años
5. Hace más de 5 años

En los ÚLTIMOS 12 MESES: ¿En cuáles de los siguientes lugares has encontrado alguna persona con la que hayas mantenido relaciones sexuales? Señala TODAS las opciones que correspondan.

1. Sex shops
2. Discotecas, clubs y bares gais
3. Saunas
4. Clubes de sexo
5. Internet
6. Apps
7. Parques, baños públicos y otros lugares de ligue o "cruising"
8. Fiesta sexual en una casa privada
9. Otro lugar: Especificar

Y ¿en cuál has encontrado EL MAYOR NÚMERO de parejas? **Transferir respuestas de la anterior**

1. Sex shops
2. Discotecas, clubs y bares gais
3. Saunas

4. Clubes de sexo
5. Internet
6. Apps
7. Parques, baños públicos y otros lugares de ligue o "cruising"
8. Fiesta sexual en una casa privada
9. Otro lugar. Especificar:

FILTRO MUJERES: A LOS QUE HAYAN CONTESTADO QUE HAN TENIDO SEXO: “Más a menudo con mujeres, pero al menos una vez con un hombre”, “Igual con hombres que con mujeres” o “Más a menudo con hombres, pero al menos una vez con una mujer”……

¿Cuándo fue la última vez que tuviste sexo con alguna mujer?

1. En el último mes
2. Últimos 6 meses
3. Últimos 12 meses
4. Últimos 5 años *[salta 1 pregunta]*
5. Hace más de 5 años *[salta 1 pregunta]*

En los ÚLTIMOS 12 MESES, ¿Con cuántas mujeres has tenido sexo sin preservativo?

1. Ninguna
2. Una
3. Menos de 5
4. Menos de 10
5. Más de 10

[Termina el filtro MUJERES. A partir de aquí, responden todos]

**SOBRE LA PRUEBA DEL VIH**

¿Cuántas veces te has hecho la prueba del VIH? [Respuesta obligatoria]

1. Nunca *[salta 3 preguntas]*
2. Una vez
3. 2 veces
4. 3 a 5 veces
5. 6 a 9 veces
6. 10 a 15 veces

7. 16 a 20 veces
8. Más de 20 veces

Y ¿cuándo te la hiciste por última vez?

1. En el último mes
2. Últimos 3 meses
3. Últimos 6 meses
4. Últimos 12 meses
5. Últimos 5 años
6. Hace más de 5 años

¿Cuál fue el resultado de la última prueba? [Respuesta obligatoria]

1. Positivo (Tengo el VIH)
2. Negativo (No tenía el VIH) [salta 1 pregunta]
3. No fui a recoger el resultado [salta 1 pregunta]

¿Cuál es exactamente tu situación HOY con respecto al VIH?

1. En tratamiento y el virus es indetectable
2. En tratamiento, pero tengo el virus detectable
3. Me están realizando pruebas, y todavía no me han puesto tratamiento
4. Me han dicho que tengo el VIH, pero todavía no me han dado cita para más pruebas
5. Di positivo en una prueba rápida y estoy esperando la confirmación
6. Me han dicho que tengo el VIH, pero no he querido hacerme más pruebas

SOBRE INFECCIONES DE TRANSMISIÓN SEXUAL

¿Te han diagnosticado alguna de las siguientes infecciones de transmisión sexual? Señala TODAS las que correspondan:

1. Sífilis
2. Gonorrea
3. Clamidia
4. Linfogranuloma venéreo
5. Verrugas anales o genitales
6. Herpes genital o anal.

7. Virus de la hepatitis (A, B o C)
8. No he sido diagnosticado de ninguna infección de transmisión sexual *[salta 2 preguntas]*

¿Y cuál fue la ÚLTIMA que te diagnosticaron? Si te diagnosticaron más de una en la misma visita, señálalas todas: (transferir respuestas anteriores)

1. Sífilis
2. Gonorrea
3. Clamidia
4. Linfogranuloma venéreo
5. Verrugas anales o genitales
6. Herpes genital o anal.
7. Virus de la hepatitis (A, B o C)

¿Cuánto hace que te diagnosticaron esta ÚLTIMA infección?

1. En el último mes
2. En los últimos 6 meses
3. En los últimos 12 meses
4. En los últimos 5 años
5. Hace más de 5 años

CONSUMO DE DROGAS (no está en el cuestionario, es para nosotros)

Vamos a hablar bastante del CONSUMO DE DROGAS. A continuación puedes ver las que se incluyen en cada grupo y diferentes nombres por las que son conocidas, por si tienes alguna duda.

Luego ya sólo pondremos el nombre que ves en negrita, aunque deberás seleccionarlo también cuando hayas consumido otra del mismo grupo.

1. **Poppers**.
2. **Viagra**® u otras que ayudan a la erección.
3. **Tranquilizantes** (Valium®, Rohipnol®, trankimazin®, etc.).
4. **Cannabis** o canabinoides sintéticos (marihuana, hachís, porros, marihuana sintética, spice, K2,).
5. **Cocaína** en polvo o en crack (farlopa, base, basuco, etc).

6. **Heroína u opiáceos** (paja de adormidera, opio, fentanilo, metadona, buprenorfina).
7. **Anfetamina/speed**.
8. **Metanfetamina** (tina, crystal meth, T).
9. **Éxtasis** o MDMA en pastillas (pastis, pirulas) o en cristalitos o polvo (M, cristal).
10. **GHB**/GBL (G, bote, éxtasis líquido).
11. **Mefedrona** (mefe) u otros estimulantes parecidos.
12. **Ketamina** (K, keta, kei).
13. **LSD** (ácido, tripis).

¿Cuándo fue la última vez que consumiste alguna de estas drogas?

1. Poppers.
2. Viagra o similares
3. Tranquilizantes
4. Cannabis
5. Cocaína (polvo o crack)
6. Heroína u opiáceos
7. Anfetamina/speed
8. Metanfetamina
9. Éxtasis
10. GHB
11. Mefedrona o similares
12. Ketamina
13. LSD
14. Alcohol

Opciones de respuesta para cada opción

1. Nunca la he consumido
2. Último més
3. Últimos 6 meses
4. Últimos 12 meses
5. Últimos 5 años
6. Más de 5 años

Si todas las respuestas son “Nunca la he consumido” → [Salta a Escalas de Dependencia]

TAMPOCO responde a inyección de drogas ni esnifar drogas.

SI responde a inyección esteroides

¿Intenta recordar cuántos años tenías la primera vez que consumiste estas drogas?

/__/__/ Transferir respuestas de los que las han consumido drogas en la vida **menos ALCOHOL**

En los ÚLTIMOS 12 MESES: ¿Aproximadamente cuántos días has consumido estas drogas?

Transferir respuestas de los que las han consumido en el último año (respuestas 2, 3,4). No transferir **NUNCA ALCOHOL**

1. Un día
2. Menos de 5
3. Menos de 10
4. Menos de 20
5. Menos de 50
6. Menos de 100
7. Más de 100

SOBRE LA PREOCUPACIÓN POR TU CONSUMO

¿Alguna vez has consultado con un profesional de salud por tu preocupación sobre el consumo de drogas?

1. Sí
2. No [Sata 2 preguntas]

¿Cuándo ha sido la última vez?

1. En el último mes
2. En los últimos 6 meses
3. En los últimos 12 meses
4. En los últimos 5 años
5. Hace más de 5 años

¿Cuál fue la droga por la que principalmente consultaste la última vez?

Lista de las drogas consumidas en cualquier circunstancia (transferir respuesta, menos VIAGRA Y ALCOHOL)

[Saltar 3 preguntas] Si la respuesta anterior fue 1, 2 o 3

Si la respuesta anterior fue 4 o 5 seguir

Pero, durante los últimos 12 meses te has sentido preocupado por tu consumo de alguna droga y has pensado que deberías consultar con un profesional sanitario?

1. Sí
2. No [Saltar 2 preguntas]

¿Cuál es la droga que te ha preocupado más?

Lista de las drogas consumidas en cualquier circunstancia (transferir respuesta, menos VIAGRA Y ALCOHOL)

¿Cuál es la razón principal por la que no has consultado, aunque hayas pensado que deberías haberlo hecho?.....

SOBRE EL CONSUMO DE ALCOHOL

Solo mostrar a aquellos que hayan **consumido alcohol en el último año** en cualquier circunstancia

¿Con qué frecuencia consumes bebidas alcohólicas?

1. Nunca [Saltar 7 preguntas]
2. Una o menos veces al mes
3. 2 a 4 veces al mes
4. 2 ó 3 veces a la semana
5. 4 o más veces a la semana

¿Cuántas bebidas alcohólicas consumes normalmente cuando bebes?

1. 1 ó 2
2. 3 ó 4
3. 5 ó 6
4. 7 a 9
5. 10 o más

¿Con qué frecuencia te tomas 6 o más bebidas alcohólicas en un solo día?

1. Nunca
2. Menos de una vez al mes
3. Mensualmente
4. Semanalmente
5. A diario o casi a diario

[Si las dos respuestas anteriores fueron la primera opción (“1 ó 2” y “Nunca”) saltar5 preguntas]

¿Con qué frecuencia, en el curso del último año, has sido incapaz de parar de beber una vez que habías empezado?

1. Nunca
2. Menos de una vez al mes
3. Mensualmente
4. Semanalmente
5. A diario o casi a diario

¿Con qué frecuencia, en el curso del último año, no pudiste atender tus obligaciones porque habías bebido?

1. Nunca
2. Menos de una vez al mes
3. Mensualmente
4. Semanalmente
5. A diario o casi a diario

¿Con qué frecuencia, en el curso del último año, has necesitado beber en ayunas para recuperarte después de haber bebido mucho el día anterior?

1. Nunca
2. Menos de una vez al mes
3. Mensualmente
4. Semanalmente
5. A diario o casi a diario

¿Con qué frecuencia, en el curso del último año, has tenido remordimientos o sentimientos de culpa después de haber bebido?

1. Nunca
2. Menos de una vez al mes
3. Mensualmente
4. Semanalmente
5. A diario o casi a diario

¿Con qué frecuencia, en el curso del último año, no has podido recordar lo que sucedió la noche anterior porque habías estado bebiendo?

1. Nunca
2. Menos de una vez al mes
3. Mensualmente
4. Semanalmente
5. A diario o casi a diario

Tú, o alguna otra persona, ¿habéis resultado heridos porque habías bebido?

1. No
2. Sí, pero no en el curso del último año
3. Sí, en el último año

¿Algún familiar, amigo, médico o profesional sanitario han mostrado preocupación por tu consumo de alcohol, o te ha sugerido que dejes de beber?

1. No
2. Sí, pero no en el curso del último año
3. Sí, en el último año

SOBRE EL CONSUMO DE DROGAS Y EL SEXO CON HOMBRES

Nos centramos ahora en el consumo de DROGAS pero SÓLO en relación con el sexo con hombres, NO en otras circunstancias.

En los últimos 12 meses, ¿qué proporción del sexo que has tenido ha sido bajo los efectos de alguna droga (considera también los poppers o viagra)?

1. Nada
2. Casi nada
3. Menos de la mitad
4. La mitad
5. Más de la mitad
6. Casi todo
7. Todo

Te vamos a presentar ahora las drogas que nos has dicho que has consumido ALGUNA VEZ en cualquier circunstancia, ¿Cuáles has tomado alguna vez en las 6 horas previas o durante el sexo anal? Señalar TODAS las que se han consumido:

Nunca he consumido drogas para sexo [Salta hasta escalas de dependencia]

(Mas las drogas consumidas en cualquier circunstancia menos el ALCOHOL)

Ahora, dinos cuáles fueron las tres PRIMERAS que consumiste PARA EL SEXO.

Pon un 1 en de la primera que utilizaste, un 2 en la segunda, y un 3 en la tercera.

Si empezaste a consumir más de una en la misma ocasión, ponlas por el orden en que las consumiste.

(Transferir respuestas de la pregunta anterior)

Ahora, dinos las tres MÁS CONSUMIDAS POR TI para el sexo.

Pon un 1 en la que más veces has consumido, un 2 en la segunda y un 3 en la tercera.

(Transferir respuestas de la pregunta consumo drogas para sexo)

De esos días que tuviste sexo anal después de consumir drogas, ¿en cuántos habías consumido ADEMÁS 6 o más vasos/copas/cañas de bebidas alcohólicas en las 6 horas previas al sexo anal?

1. Ninguno
2. Menos de la mitad
3. Aproximadamente la mitad
4. Más de la mitad
5. Todos

PARA CADA DROGA CONSUMIDA para sexo (todo el bloque que viene) PERO:

- **No hacerlo aunque lo hayan consumido: sedantes, LSD o alcohol**
- **Para el cannabis y la heroína únicamente las preguntas g y g1**
- **Para los POPPERS, LA VIAGRA, únicamente la pregunta g**

Con respecto a… (droga abreviada)

a) la PRIMERA VEZ que la consumiste en las 6 horas previas o durante el sexo anal

1. Era la primera vez que probaba esa sustancia [Salta 1 pregunta]
2. La había tomado ya antes de emplearla para el sexo

Droga abreviada (en cada pregunta del bloque)

b) ¿Cuántos días la habías consumido antes de emplearla para el sexo?

1. Uno
2. Menos de 5
3. Menos de 10
4. Menos de 20
5. Menos de 50
6. Menos de 100
7. Más de 100

c) Y esa primera vez que la usaste para sexo anal…

1. La tomé precisamente para que me hiciera efecto durante el sexo
2. No la tomé pensando en tener sexo, pero lo tuve estando bajo su efecto

d) Y esa primera vez, esa sustancia

1. La tenía yo antes de quedar para tener sexo
2. Me la dieron en el lugar en el que tuve sexo
3. La compré en el lugar en el que tuve sexo

e) Y en esa ocasión, tuviste sexo con:

1. Sólo mi pareja estable [Salta 1 pregunta]
2. Mi pareja estable y una pareja ocasional
3. Mi pareja estable y dos o más ocasionales
4. Una pareja ocasional
5. Dos o más parejas ocasionales

f) Y utilizaste el condón con

1. Todos
2. Con las ocasionales pero no con mi pareja estable
3. Ni con las ocasionales ni con la estable

g) Y, ¿Cuánto hace que has consumido esta sustancia por última vez para tener sexo?

1. En el último mes
2. En los últimos 6 meses

3. En los últimos 12 meses
4. En los últimos 5 años [no preguntar pregunta g1]
5. Hace más de 5 años [no preguntar pregunta g1]

En cocaína, anfetamina, metanfetamina, éxtasis, ghb, mefedrona, ketamina:

g.1) En los ÚLTIMOS 12 MESES: cuando has consumido esta sustancia ha sido

1. Siempre justo o antes o durante el sexo
2. La mayoría de las veces justo antes o durante el sexo
3. La mitad de las veces justo antes o durante el sexo.
4. Pocas veces en relación en el sexo.

g.2) Y EN TODA TU VIDA, ¿Cuántos hombres que nunca habían consumido esta sustancia comenzaron a consumir cuando tuvieron sexo contigo porque se lo propusiste?

1. Ninguno
2. Uno
3. Menos de 5
4. Menos de 10
5. Más de 10

g.3) Y cuántas mujeres**: **(No a los que digan que sólo han mantenido relaciones sexuales con hombres)

1. Ninguna
2. Una
3. Menos de 5
4. Menos de 10
5. Más de 10

SOBRE SESIONES DE SEXO Y CONSUMO DE DROGAS

Las siguientes preguntas se refieren EXCLUSIVAMENTE a SESIONES de sexo y consumo de drogas, es decir, cuando intencionadamente se consumen drogas para tener relaciones sexuales especiales (de más duración, con más personas, haciendo cosas distintas, etc.)..

¿Has participado en alguna ocasión en sesiones de este tipo?

1. Sí
2. No [Salta siguiente sección]

¿Cuánto hace que lo hiciste por última vez?

1. Menos de una semana
2. Menos de un mes
3. Menos de 3 meses
4. Menos de 6 meses
5. Menos de un año
6. Más de un año

¿En cuántas ocasiones después de una sesión has tomado profilaxis post-exposición?
(pastillas que se toman tras tener sexo que podría transmitir el VIH y que reducen la probabilidad de infectarse).

1. Ninguna
2. Una o dos
3. De 3 a 5
4. De 6 a 10
5. De 10 a 20
6. Más de 20

Aparte de la profilaxis post-exposición ¿Alguna vez has tenido que recibir asistencia médica de urgencia durante o después de una sesión?

1. Sí
2. No [Salta 2 preguntas]

¿Cuántas veces?

1. Una
2. Dos
3. Tres
4. Cuatro
5. Cinco o más

¿Cuál fue el motivo principal por el que consultaste la última vez? ....

[Salta 2 preguntas]

¿Aunque no llegaras a consultar, crees que deberías haberlo hecho durante o después de alguna sesión?

1. Sí
2. No [Salta 1 pregunta]

¿Qué te sucedió la última vez que crees que deberías haber consultado? ………………………..

¿Cuántas horas duró la sesión más larga que has tenido?

/__/__/ (Sólo valores numéricos).

¿Cuál ha sido el mayor número de hombres con los que has tenido sexo sin preservativo en una misma sesión?

/__/__/ (Sólo valores numéricos).

¿Has realizado o te han hecho realizar en una sesión alguna práctica de riesgo que no habías hecho nunca antes?

1. Sí
2. No [Salta 1 pregunta]

¿Cuáles han sido esas prácticas de riesgo que nunca antes habías realizado?
Puedes señalarnos *hasta tres, ordenadas según la preocupación que te causaron.*

1. …..
2. …..
3. …..

Los que respondieron consumo de drogas en cualquier circunstancia en los últimos 12 meses (respuestas 2, 3 y 4 de la primera pregunta de drogas añadir el módulo de la escala de dependencia.
Hacerlo para todas las drogas excepto: sedantes y LSD. Tampoco poppers, viagra, cannabis y alcohol.

SOBRE TU VALORACIÓN DE TU CONSUMO DE DROGAS Y TU ACTIVIDAD SEXUAL

a. **Piensa sobre esta frase: "LO HICE DEMASIADO". [Respuesta obligatoria]**

En los últimos 12 meses, con qué frecuencia esta frase se aplica a tu actividad sexual o a tu consumo de algunas drogas (con cualquier finalidad, no sólo para sexo):

Escala de respuestas:

1. Nunca
2. Muy pocas veces
3. Algunas veces
4. Casi siempre
5. Siempre
6. No sé /prefiero no contestar

FILAS:

1. Actividad sexual excesiva o uso inapropiado de pornografía (online u offline).
2. Respuestas transferidas de las drogas consumidas en cualquier circunstancia ultmos 12 meses

b. Piensa sobre esta frase: "CUANDO EMPEZABA NO PODÍA PARAR". [Respuesta obligatoria]

En los últimos 12 meses, con qué frecuencia esta frase se aplica a

c. Piensa sobre esta frase: "SENTÍA QUE TENÍA QUE HACERLO PARA FUNCIONAR" [Respuesta obligatoria]

En los últimos 12 meses, con qué frecuencia esta frase se aplica a

d. Piensa sobre esta frase: "SEGUÍ HACIÉNDOLO, AUNQUE ME CAUSÓ PROBLEMAS" [Respuesta obligatoria]

En los últimos 12 meses, con qué frecuencia esta afirmación se aplica a

SOBRE LA INYECCIÓN DE ESTEROIDES Y OTRAS SUSTANCIAS

¿Alguna vez te has inyectado (o te han inyectado) ESTEROIDES anabolizantes, como testosterona o similares? [Respuesta obligatoria]

1. Sí
2. No *[salta 3 preguntas]*

¿Y cuándo lo has hecho por última vez?

1. En el último mes
2. En los últimos 6 meses
3. En los últimos 12 meses
4. En los últimos 5 años
5. Hace más de 5 años

¿Qué edad tenías la primera vez que sucedió?

/__/__/

Por COMPARTIR entendemos usar una jeringuilla que ya ha usado otro, tomar las sustancias disueltas de la jeringuilla usada por otro o del frasco o recipiente donde había metido la jeringuilla usada otra persona.

Teniendo esto en cuenta, ¿Alguna vez has COMPARTIDO al inyectarte esteroides?

1. Sí
2. No

¿Alguna vez te has inyectado (o te han inyectado) alguna DROGA PARA COLOCARTE? Da igual si fue en relación con el sexo o no.

1. Sí
2. No *[salta siguiente sección]*

¿Y cuándo lo has hecho por última vez?

1. En el último mes
2. En los últimos 6 meses
3. En los últimos 12 meses
4. En los últimos 5 años
5. Hace más de 5 años

Qué edad tenías la primera vez que sucedió?

/__/__/

¿Te la inyectaste tú o te la inyectó otra persona?

1. Yo mismo *[salta 1 pregunta]*
2. Otra persona

¿Quién era esa otra persona?

1. Pareja sexual estable
2. Pareja sexual ocasional
3. Hermano
4. Otro familiar
5. Amigo muy cercano
6. Traficante o camello
7. Conocido casual
8. Otras personas. Especificar:

¿Y qué droga te inyectaste o te inyectaron esa primera vez?

Respuestas transferidas de las drogas consumidas en cualquier circunstancia

1. Cocaína (polvo o crack)
2. Heroína u opiáceos
3. Anfetamina/speed
4. Metanfetamina
5. Éxtasis
6. Mefedrona o similares
7. Ketamina

Y, esa primera vez que te inyectaste, ¿lo hiciste en las 6 horas previas o durante el sexo anal)?

1. Sí
2. No

Y EN TODA TU VIDA ¿cuántos días te has inyectado (o te han inyectado) alguna droga para colocarte?

1. Uno
2. Menos de 5
3. Menos de 10
4. Menos de 20
5. Menos de 50
6. Menos de 100

7. Más de 100

De las siguientes drogas, ¿cuáles te has inyectado (o te han inyectado) alguna vez en tu vida?

[multiple respuesta] Respuestas transferidas de las drogas consumidas en cualquier circunstancia

1. Cocaína (polvo o crack)
2. Heroína u opiáceos
3. Anfetamina/speed
4. Metanfetamina
5. Éxtasis
6. Mefedrona o similares
7. Ketamina

Ahora, dinos las tres que te has inyectado (o te han inyectado) MÁS VECES. De mayor a menor frecuencia.

Pon un 1 en la droga que más veces te has inyectado, un 2 en la segunda y un 3 en la tercera.

(Transferir respuestas de la pregunta anterior)

Y, ¿Te has inyectado drogas para colocarte alguna vez en las 6 horas previas o durante el sexo anal?

1. Sí
2. No *[salta 1 pregunta]*

Y, ¿Cuál ha sido la droga que te has inyectado más veces para el sexo anal?

[Listar sólo las que hay dicho que se ha inyectado

Por COMPARTIR entendemos usar una jeringuilla que ya ha usado otro, tomar las sustancias disueltas de la jeringuilla usada por otro o del frasco o recipiente donde había metido la jeringuilla usada otra persona.

Teniendo esto en cuenta, ¿Alguna vez has COMPARTIDO al inyectarte drogas para colocarte?

1. Sí
2. No *[salta 3 preguntas]*

¿Y cuándo lo has hecho por última vez?

1. En el último mes
2. En los últimos 6 meses
3. En los últimos 12 meses
4. En los últimos 5 años
5. Hace más de 5 años

Y en TU VIDA, ¿con cuántas personas distintas crees que habrás COMPARTIDO al inyectarte drogas para colocarte?

1. Una
2. Menos de 5
3. Menos de 10
4. Menos de 20
5. Menos de 50
6. Más de 50

¿Y con cuántas de ellas ha sido para mantener relaciones sexuales?

1. Ninguna
2. Menos de la mitad
3. La mitad
4. Más de la mitad
5. Todas

ALGO MÁS SOBRE TI

¿Cuántos habitantes tiene la localidad en la que vives?

1. Más de un millón
2. Más de 500 mil
3. Más de 100 mil
4. Más de 50 mil
5. Más de 10 mil
6. Menos de 10 mil

Tu situación económica es:

1. Muy cómoda
2. Cómoda, está bien
3. Es ajustada, tengo que tener cuidado para llegar a fin de mes
4. Llego a final de mes con dificultad
5. Llego a final de mes con deudas

FILTRO: LOS QUE NO HAYAN NACIDO EN ESPAÑA PERO VIVAN EN ESPAÑA responden pregunta:

¿Cuántos años llevas viviendo en España? (2 dígitos)

/__/__/ ***Pon "0" años si llevas menos de uno***

SOBRE EL AUTOTEST DE VIH

Vamos a hablar del autotest de VIH, es decir, cuando la misma persona interesada en conocer si tiene el VIH, utiliza un kit para tomarse la muestra, realizarse la prueba y obtener su resultado en 15-20 minutos, sin la ayuda de ningún profesional.

¿Sabías que en ESPAÑA desde hace ya más de un año, puedes comprar un autotest de VIH en la farmacia y parafarmacia sin necesidad de receta médica?

1. No, no lo sabía [Salta siguiente sección]
2. Algo había oído, pero no estaba seguro
3. Sí, lo sabía

¿Cómo te enteraste?

1. Por una pareja sexual
2. Por amigos/as
3. En una asociación LGTB
4. En una asociación de VIH/SIDA
5. En una página web de contactos para gais
6. A través de medios de comunicación orientados a la comunidad gay
7. En medios de comunicación generales
8. A través de una página web relacionada con el VIH
9. Lo vi en una farmacia
10. Lo vi anunciado en una farmacia en internet
11. Lo vi en una máquina expendedora

12. Otra forma, especificar

FILTRO: solo siguen los que marcaron que si lo sabían

Los que algo habían oído, pero no estaban seguro, (respuesta 2) → a PREP

Te HAS HECHO alguna vez la prueba del VIH usando un AUTOTEST?

1. Sí
2. No [Salta 5 preguntas]

¿Cuántas veces?

1. Una vez
2. Dos
3. Tres o más

¿Cuándo lo utilizaste por última vez? Hace:

1. Menos de 3 meses
2. De 3 a 6 meses
3. De 6 meses a 1 año
4. De uno a 2 años
5. De 2 a 5 años
6. Más de 5 años

¿Cómo conseguiste este ÚLTIMO AUTOTEST?.

1. Lo compré en una farmacia en España
2. Lo compré en una farmacia online en España
3. Lo compré en una máquina expendedora en España.
4. Lo compré en un país donde se vende legalmente (por internet, en una farmacia, parafarmacia, etc.)
5. Lo compré por internet sin saber si era legal o no
6. Otra forma. Especificar:

¿Estabas solo o acompañado cuando te lo hiciste?

1. Solo [Salta 1 pregunta]

2. Acompañado

¿Quién te acompañaba?

1. Mi pareja estable
2. Una pareja ocasional
3. Un amigo/a
4. Un familiar
5. Personal de una ONG
6. Personal sanitario
7. Otra persona, especificar:

[Salta 3 pregunta]

¿Te has planteado seriamente alguna vez utilizar el autotest en vez de hacerte la prueba en un centro o unidad móvil?

1. Sí
2. No [Salta 1 pregunta]

¿Cuál ha sido la principal razón por la que finalmente decidiste no utilizarlo?

1. Obtener la muestra, realizar la prueba e interpretar los resultados es algo que debe hacer un profesional
2. La presencia de un profesional que te asesore y que te comunique el resultado son imprescindibles
3. No confío en la calidad de la prueba, tengo dudas sobre la validez de los resultados
4. No me compensa pagar los 25€ que cuesta, pues puedo hacérmela en muchos lugares sin pagar
5. Otra. Especificar

[Salta 1 pregunta]

¿Cuál ha sido la principal razón por la que nunca te has planteado seriamente utilizarlo?

1. Obtener la muestra, realizar la prueba e interpretar los resultados es algo que debe hacer un profesional
2. La presencia de un profesional que te asesore y que te comunique el resultado son imprescindibles
3. No confío en la calidad de la prueba, tengo dudas sobre la validez de los resultados

4. No me compensa pagar los 25€ que cuesta, pues puedo hacérmela en muchos lugares sin pagar
5. Otra. Especificar

**SOBRE la PREP**

SECCIÓN PARA VIH NEGATIVOS (para nosotros, no poner en el cuestionario)

(La PrEP es una estrategia que consiste en la que las personas VIH-negativas toman medicamentos antirretrovirales para disminuir su riesgo de infectarse con este virus).

¿Alguna vez has tomado la PrEP?

1. Sí
2. No [Salta al final del cuestionario]

¿Cómo la has tomado?

1. Cuando la he necesitado [Sata 3 preguntas]
2. Algunas veces cuando la he necesitado, pero también diariamente en algún período
3. La tomé diariamente, pero ya no lo hago
4. La estoy tomando diariamente [Salta 1 pregunta]

¿En total, durante cuánto tiempo la has tomado diariamente en tu vida?

1. Menos de un mes
2. Menos de 3 meses
3. Menos de 6 meses
4. Menos de un año
5. Más de un año

[Salta 1 pregunta]

¿Desde cuándo llevas tomándola diariamente?

1. Menos de un mes
2. Menos de 3 meses
3. Menos de 6 meses
4. Menos de un año
5. Más de un año

¿En qué medida el poder tener sesiones de sexo bajo el efecto de drogas y sin usar el preservativo ha sido una razón importante para tomar Prep?

1. Muy importante
2. Bastante importante
3. No estoy seguro
4. Poco importante
5. Nada importante.

[Salta al final del cuestionario]

Si la respuesta a toma de prep es 1 ó 2 : 169

¿Qué proporción de las veces que la has tomado de forma no diaria ha sido para tener sesiones de sexo bajo el efecto de drogas sin tener que usar el preservativo?

1. Siempre
2. La mayoría de las veces
3. La mitad
4. Menos de la mitad
5. Nunca

[Salta al final del cuestionario]

**SOBRE la PREP**

SECCIÓN PARA VIH POSITIVOS (para nosotros, no poner en el cuestionario)

(La PrEP es una estrategia que consiste en la que las personas VIH-negativas toman medicamentos antirretrovirales para disminuir su riesgo de infectarse con este virus).

¿Alguna vez tomaste la PrEP?

1. Sí
2. No [Salta al final del cuestionario]

¿Cómo la tomaste?

1. Cuando la necesité, nunca diariamente [Salta 2 preguntas]
2. Algunas veces cuando la necesité, pero también diariamente en algún período
3. Sólo diariamente

¿En total, durante cuánto tiempo la tomaste diariamente en tu vida?

1. Menos de un mes

2. Menos de 3 meses
3. Menos de 6 meses
4. Menos de un año
5. Más de un año

¿En qué medida el poder tener sesiones de sexo bajo el efecto de drogas y sin usar el preservativo ha sido una razón importante para tomar Prep?

1. Muy importante
2. Bastante importante
3. No estoy seguro
4. Poco importante
5. Nada importante

Final del cuestionario solo en respuesta 3 ¿Cómo la tomaste?

Si la respuesta a toma de prep es 1 ó 2 :

¿Qué proporción de las veces que la has tomado de forma no diaria ha sido para tener sesiones de sexo bajo el efecto de drogas sin tener que usar el preservativo?

3. Siempre
4. La mayoría de las veces
5. La mitad
6. Menos de la mitad
7. Nunca

¡¡¡¡¡¡¡¡¡¡¡MUCHAS GRACIAS por tu cooperación¡¡¡¡¡¡¡¡¡¡¡¡

Queremos conocer la experiencia del mayor número de hombres que tienen relaciones sexuales con hombres . Por eso, por favor, copia el link que tienes debajo y envíaselo por e-mail, Whatsapp, Instagram u otro medio a tus amigos para que podamos contar también con su experiencia. Anímalos a que contesten.

https://es.surveymonkey.com/r/online_rds
