# Supplementary material for: Excessive Drinking Among Men Who Have Sex With Men Recruited From Web-Based Resources: Cross-sectional Questionnaire Study
Source: JMIR Public Health Surveill. 2022 Oct 31;8(10):e32888. doi: 10.2196/32888 (PMC9664322; doi:10.2196/32888)
Supplement: Multimedia Appendix 1 [file publichealth_v8i10e32888_app1.zip › MSM sample questionnaire_Smartphone_English.pdf]

Presentation Screen 1A:

We are researchers at the Instituto de Salud Carlos III. We are carrying out a study funded by the Ministry of Health, addressed to men who have sex with men. Our goal is to characterize recreational or sex-related substance use and to identify common health risks, prevention measures and healthcare requirements. This study has received approval by the Ethical Committee of our institution.

This survey is completely anonymous. We do not collect IP directions, nor other information driving to personal identification.

Please participle only once, even if you receive the invitation to participate by several ways.

It will take 10-15 minutes.

Presentation Screen 1B:

This questionnaire is more easily fulfilled in a tablet or computer. Thus:

- If you are using a tablet or a computer, please click on this link: https://es.surveymonkey.com/r/online_v4
- If you are using a **smartphone**:
  - To shift to a tablet or computer, please copy and paste the previous link and send it to your own mail address.
  - If you want to continue using your smartphone, please click here :
  - https://es.surveymonkey.com/r/onlinemovil_v3

Screen 2, Introduction: Smartphone version

To participate, please confirm the following requisite:

Para participar por favor, marca la siguiente casilla

☐ On my age, I am legally authorized to have sexual relations and I want to participate in the study.

Before to start **REMEMBER**:

- To advance in the questionnaire use the buttons PREVIOUS and NEXT that are placed after each question.

- **DO NOT** use the “back” button in your smartphone, doing so you may abandon the survey. If so, refresh the site ↻ and you will obtain a message. Press **continue** to go return to the survey.

You are... Q1. [Answering is mandatory]

1. Man
2. Woman
3. Trans

[If Q1=2 OR 3] **Q1b. This study is addressed to men. You are invited to read and fulfil the rest of the survey, however, we will not use your responses. What would you like to do?**

1. Leave the survey
2. Continue reading

[If Q1b=1] Thank you very much for your interest in our project

¿Where you were born?

1. Spain
2. Other----- Specify: _________________

¿How old are you? [Answering is mandatory]

/__/__/ If less than 16 years → You are not legally authorized to have sexual relations and thus you are not allowed to participate in the study.

¿In which country do you live?

3. Spain
4. Other----- Specify: _________________[skip 1 question]

¿And in which province? **List of provinces**

Please do not leave blank any question, please select the answer you find the most appropriate, even if you are not sure

¿Which is the highest education level you have reached?

1. None or primary education (until 12 years old)
2. First and Secondary Cicle of Secondary Education (until 16 years old)
3. High Secondary School; Medium Grade Professional Training (until 18 years old)
4. High Grade Professional Training (until 20 years old)
5. University. Grade, Master, Doctorate

ABOUT SEXUALITY

The term "sexual relations" or "having sex" can be used differently. Here we refer exclusively to anal or vaginal intercourse.

Following the previous definition, in your life course, have you had sexual relations?

[Answering is mandatory]

1. No, never, neither with men nor with women [Leave the survey]
2. Only with women [Leave the survey]
3. More frequently with women, but at least once with men [skip 1 question] *(filter women)*
4. In the same frequency with men and with women [skip 1 question] *(filter women)*
5. More frequently with men, but at least once with women [skip 1 question] *(filter women)*
6. Only with men [skip 1 question] *(filter: do not display questions referring to women)*

This questionnaire is addressed exclusively to men who have had sex with men and only if anal intercourse has had existed.

You are invited to read and fulfil the rest of the survey, however, we will not use your responses. What would you like to do?

1. Leave the survey
2. Continue reading

Among the following, which option describes better the way you live your sexual life with other men?

1. Openly
2. Discretly
3. Hidely
4. Unconfessed completely

Remember “sexual relations” or “having sex”: ONLY if anal or vaginal intercourse.

¿How old were you when you had your first sexual relation with another man? /__/__/

IN THE LAST 12 MONTHS ¿with how many men have you had sexual relations without preservative?

1. None
2. One
3. Less than 5
4. Less than 10
5. Less than 20
6. Less than 50
7. Less than 100
8. Less than 200
9. More than 200

In your life course, how many times have you paid another man to have sex with you? As “pay” we refer to giving money, gifts or favors in exchange of sex.

1. None [skip 1 question]
2. One
3. Less than 5
4. Less than 10
5. Less than 20
6. Less than 50
7. Less than 100
8. More than 100

When was last time?

1. Last month
2. In the last 6 months
3. In the last 12 months
4. In the last 5 years
5. More than 5 years ago

In your life course, how many times have you been paid by another man in order to have sex with him?

1. None [skip 1 question]
2. One
3. Less than 5
4. Less than 10
5. Less than 20
6. Less than 50
7. Less than 100
8. More than 100

How old were you the first time?

/__/__/

When was last time?

1. Last month
2. In the last 6 months
3. In the last 12 months
4. In the last 5 years
5. More than 5 years ago

IN LAST 12 MONTHS: In which of the following have you found any person with which you have had sexual relations? Select ALL that apply.

1. Sex shops
2. Night clubs and bars
3. Saunas
4. Sex clubs
5. Internet
6. Apps
7. Parks, public toilets and other "cruising" places
8. Sexual parties at private households
9. Other place: Specify

And in which of them have you found THE HIGHEST NUMBER of partners? **Transfer answers from previous question**

1. Sex shops
2. Night clubs and bars
3. Saunas
4. Sex clubs
5. Internet
6. Apps
7. Parks, public toilets and other "cruising" places
8. Sexual parties at private households
9. Other place: Specify

FILTER WOMEN: FOR THOSE WHO HAVE ANSWERED: "More frequently with women, but at least once with men", "In the same frequency with men and with women" o "More frequently with men, but at least once with women"……

When was the last time you had sex with a woman?

1. Last month
2. In the last 6 months
3. In the last 12 months
4. In the last 5 years [skip 1 question]
5. More than 5 years ago [skip 1 question]

In LAST 12 MONTHS, with how many women have you had sexual relations without preservative?

1. None
2. One
3. Less than 5
4. Less than 10
5. More than 10

[Filter women has finished. Onwards, all participants are invited to answer]

ABOUT HIV TEST

How many times have you been tested for HIV? [Answering is mandatory]

1. Never *[skip 3 questions]*
2. Once
3. 2 times
4. 3 to 5 times
5. 6 to 9 times
6. 10 to 15 times
2. 16 to 20 times
3. More than 20 times

When was last time?

1. Last month
2. In the last 3 months
3. In the last 6 months
4. In the last 12 months
5. In the last 5 years
6. More than 5 years ago

What was the result of your last test? [Answering is mandatory]

1. Positive (I've contracted HIV)
2. Negative (I've not contracted HIV) [skip 1 question]
3. I did not pick up my result [skip 1 question]

Which is exactly your CURRENT situation concerning HIV?

1. Under treatment, and "undetectable" virus
2. Under treatment, but virus still "detectable"
3. Under medical examination (diagnostic tests), and without treatment by the moment
4. I've been informed I have HIV, but I've not other medical appointments to come
5. I've been tested positive in a rapid test, and I am waiting for diagnostic confirmation
6. I've been informed I have HIV, but I don't want to continue being examined

ABOUT SEXUAL-ACQUIRED INFECTIONS

Have you ever been tested positive for any of the following sexual-acquired infections? Mark ALL that apply:

1. Syphilis
2. Gonorrhea
3. Chlamydia
4. Lymphogranuloma venereum
5. Anal or genital warts
6. Anal or genital herpesvirus
7. Hepatitis virus (A, B or C)
8. I've been never tested positive for a sexual-acquired infection *[skip 2 questions]*

And when was the last time you were diagnosed? If you were diagnosed for more than one infection in the same consultation, mark all: (transfer previous responses)

1. Syphilis
2. Gonorrhea
3. Chlamydia
4. Lymphogranuloma venereum
5. Anal or genital warts
6. Anal or genital herpesvirus
7. Hepatitis virus (A, B or C)

When was the last time you were diagnosed?

1. Last month
2. Last 6 months
3. Last 12 months
4. Last 5 years
5. More than 5 years ago

ABOUT SUBSTANCE USE

We are going to talk about **SUBSTANCE USE**. You can see below which drugs are included in each group and different terms we utilize to name them, just in case you have any doubt.

After that we will employ exclusively the term in bold. You shall select it as well if you have taken any drug from the same group.

1. **Poppers**.
2. **Viagra**® u others for erectile dysfunction.
3. **Tranquilizers** (Valium®, Rohipnol®, trankimazin®, etc.).
4. **Cannabis** or synthetic cannabinoids (marijuana, hash, synthetic marijuana, spice, K2).
5. **Cocaine** in powder or in crack (*farlopa*, *base*, *basuco*, etc).
6. **Heroine or opioids** (*paja de adormidera*, fentanyl, metadone, buprenorfine).
7. **Amphetamines/speed**.
8. **Methamphetamine** (*tina*, crystal meth, T).
9. **Ecstasy** or MDMA in pills (*pastis*, *pirulas*) or in powder (M, *cristal*)
10. **GHB**/GBL (G, *bote*, liquid ecstasy)
11. **Mephedrone** (*mefe*) or other stimulants
12. **Ketamine** (K, *keta*, *kei*)
13. **LSD** (acid, *tripis*)

When was the last time you consumed any of these drugs?

1. Poppers.
2. Viagra or similar
3. Tranquilizers
4. Cannabis
5. Cocaine (powder or crack)
6. Heroine or opioids
7. Amphetamine/speed
8. Methamphetamine
9. Ecstasy
10. GHB
11. Mephedrone or similar
12. Ketamine
13. LSD

14. Alcohol

Response alternatives for each option

1. I've never consumed it
2. Last month
3. Last 6 months
4. Last 12 months
5. Last 5 years
6. More than 5 years ago

If all responses correspond to "I've never consumed it" → [Skip to Dependence Scales]

He does not answer to injecting drugs, nor to snorting.

He responds to injecting steroids

How old were you the first time you consumed any of these drugs?

/__/__/ Transfer responses of those who have ever consumed (excepting ALCOHOL)

In LAST 12 MONTHS: Approximately, how many days have you consumed these drugs?

Transfer responses of those who have consumed in last year (answers 2, 3, 4). Do not transfer alcohol NEVER

1. One day
2. Less than 5 days
3. Less than 10 days
4. Less than 20 days
5. Less than 50 days
6. Less than 100 days
7. More than 100 days

ABOUT YOUR OWN CONCERN REGARDING SUBSTANCE USE

Have you ever consulted a health professional because of your own concern regarding substance use?

1. Yes
2. No [Skip 2 questions]

When was last time?

1. Last month
2. Last 6 months
3. Last 12 months
4. Last 5 years
5. More than 5 years ago

Because of which substance did you consult last time?

List of consumed substances in any circumstance (transfer answer, excepting VIAGRA and ALCOHOL)

[Skip 3 questions] If last answer was 1, 2 or 3

If last answer was 4 or 5, continue

During last 12 months have you ever been concerned because of your consumption of any drug, and have you considered consulting a health professional?

1. Yes
2. No [Skip 2 questions]

Because of which substance have you been concerned the most?

List of consumed substances in any circumstance (transfer answer, excepting VIAGRA and ALCOHOL)

Which is the main reason leading you not to consult, even if you considered that you had to?

You can say you response by your microphone instead of typing

ABOUT ALCOHOL USE

Only display for those who have consumed alcohol in last year

How often do you have a drink containing alcohol?

1. Never [Skip to Qs 9-10]
2. Monthly or less
3. 2 to 4 times a month

4. 2 to 3 times a week
5. 4 or more times a week

How many drinks containing alcohol do you have on a typical day when you are drinking?

1. 1 or 2
2. 3 or 4
3. 5 or 6
4. 7, 8, or 9
5. 10 or more

How often do you have six or more drinks on one occasion?

6. Never
7. Less than monthly
8. Monthly
9. Weekly
10. Daily or almost daily

[If last two responses were the first option (“1 or 2” and “Never”), skip 5 questions]

How often during the last year have you found that you were not able to stop drinking once you had started?

1. Never
2. Less than monthly
3. Monthly
4. Weekly
5. Daily or almost daily

How often during the last year have you failed to do what was normally expected from you because of drinking?

1. Never
2. Less than monthly
3. Monthly
4. Weekly
5. Daily or almost daily

How often during the last year have you needed a first drink in the morning to get yourself going after a heavy drinking session?

1. Never
2. Less than monthly
3. Monthly
4. Weekly
5. Daily or almost daily

How often during the last year have you had a feeling of guilt or remorse after drinking?

1. Never
2. Less than monthly
3. Monthly
4. Weekly
5. Daily or almost daily

How often during the last year have you been unable to remember what happened the night before because you had been drinking?

1. Never
2. Less than monthly
3. Monthly
4. Weekly
5. Daily or almost daily

Have you or someone else been injured as a result of your drinking?

1. No
2. Yes, but not in the last year
3. Yes, during the last year

Has a relative or friend or a doctor or another health worker been concerned about your drinking or suggested you cut down?

1. No
2. Yes, but not in the last year
3. Yes, during the last year

ABOUT SUBSTANCE USE AND SEX WITH OTHER MEN

We focus now on substance use but ONLY if sex-related, no under other circumstances

In the last 12 months, what proportion of your sex encounters have been under the effect of any drug (take also poppers or viagra in consideration)?

1. None
2. Almost none
3. Less than half
4. A half
5. More than half
6. Almost all
7. All

We display now those substances that you have declared to have EVER consumed in any circumstance. Which of them have you had in the previous 6 hours of an anal intercourse? Mark ALL of them:

I have never consumed any substance during a sex relation [Skip until dependence scales]

Tell us which substances were the three first you consumed during a sex relation.

Write “1” in the first one, “2” in the second one, and “3” in the third.

If your first use included more than one substance, write the order in which you took them.

(Transfer responses from last question)

Now tell us which three substances you consume more frequently during sex

Write “1” in the first one, “2” in the second one, and “3” in the third.

(Transfer responses from the question about substance use for sex)

Among those days when you had anal intercourse after consuming drugs, in how many of them have you ALSO had 6 or more glasses/cups containing alcoholic beverages in the previous 6 hours of anal sex?

1. None
2. Less than half
3. A half

4. More than half
5. All

FOR EACH DRUG CONSUMED for sex (next grill) BUT:

- **Do not display even if they have consumed: tranquilizers, LSD or alcohol**
- **For cannabis and heroine, only questions g and g1**
- **For POPPERS and VIAGRA, only question g**

Concerning… (abbreviated substance name)

a) The FIRST TIME you consumed it in previous 6 hours or during anal sex

1. It was the first time I had this substance [Skip 1 question]
2. I had already had it before taking it for sex

Abbreviated substance name (in each question of the grill)

b) How many days had you consumed it before using it for sex?

1. One
2. Less than 5
3. Less than 10
4. Less than 20
5. Less than 50
6. Less than 100
7. More than 100

c) And that time when you had it for the first time for anal sex…

1. I took it precisely to feel its effects during sex
2. I did not take it thinking on sex, but I had sex under its effects

d) And that first time, that drug…

1. I had it before meeting somebody to have sex
2. I was given it at the place where I had sex
3. I bought it at the place where I had sex

e) And that time, you had sex with:

1. Only with my current partner [Skip 1 question]

2. With my current partner and another person
3. With my current partner and two or more other people
4. With another person (not my current partner)
5. With two or more other people

f) And you used a preservative…

1. With all the other people
2. Only with people different from my current partner
3. With nobody

g) When was the last time you had that substance for having sex?

1. Last month
2. In the last 6 months
3. In the last 12 months
4. In the last 5 years [do not display question g1]
5. More tan 5 years ago [do not display question g1]

For cocaine, amphetamine, metamphetamine, ecstasy, ghb, mephedrone, ketamine:

g.1) In LAST 12 MONTHS: when you have consumed this substance was…

1. Always just before or during sex
2. Most of times just before or during sex
3. Half of times just before or during sex
4. Few times just before or during sex

g.2) And IN ALL YOUR LIFE, how many men who had never taken this substance began to do it after having sex with you?

1. None
2. One
3. Less than 5
4. Less than 10
5. More than 10

g.3) And how many women**: **(Do not display to those having said they have not had any sex relation with women)

1. None

2. One
3. Less than 5
4. Less than 10
5. More than 10

ABOUT SEX SESSIONS AND SUBSTANCE USE

The following questions concern EXCLUSIVELY sex SESSIONS and substance use, we want to say, when substances are consumed explicitly to make sex different (longer in time, involving more people, engaging in different activities, etc.)

Have you ever participated in a session like that?

1. Yes
2. No [Skip until next section]

When was the last time you did it?

1. Last week
2. Last month
3. Less than 3 months ago
4. Less than 6 months ago
5. Less year
6. More than one year ago

How many times after a session have you had post-exposition prophylaxis?

(pills that are taken after having sex with people who can potentially transmit HIV and which reduce the risk of becoming infected)

1. None
2. One or two
3. From 3 to 5
4. From 6 to 10
5. From 10 to 20
6. More than 20

Leaving apart post-exposition prophylaxis, have you ever had to go under urgent medical assistance during or after a session?

1. Yes
2. No [Skip 2 questions]

How many times?

1. One
2. Two
3. Three
4. For
5. Five or more

What was the reason for consultation last time?...

[Skip 2 questions]

Even if at the end you did not consult, do you think you should have do it any time during or after a session?

1. Yes
2. No [Skip 1 question]

What happened to you the last time you consider you should have consulted?.................

How many hours long was the longest session you have participated in?

/__/__/ (Only numeric values).

What is the highest number of men you have had sex with without preservative in the same session?

/__/__/ (Only numeric values).

Have your done or been obliged to do during a session any risky practice you had never done before?

1. Yes
2. No [Skip 1 question]

What were those risky practices?

You can write up to three, ordered by the level of concern they produced on you.

1. …..
2. …..
3. …..

ABOUT YOUR OWN VIEW CONCERNING SUBSTANCE USE AND SEX

We propose to you four sentences concerning your sexual activities and substance use IN ALL CIRCUMSTANCES (not only for sex).

Take always in consideration ONLY THE LAST 12 MONTHS

a**. Think about this sentence:**

"I DID IT TOO MUCH".

How often this sentence may be applied to an excessive sexual activity or to an unappropriated use of pornography (online or offline)

a. Never
b. Very few times
c. Sometimes
d. Almost always
e. Always
f. I don't know / I prefer not to answer

("I DID IT TOO MUCH")

How often this sentence may be applied to your use of **: (substances consumed in any circumstance, excepting poppers, Viagra, tranquilizers, cannabis, LSD and alcohol).**

a. Never
b. Very few times
c. Sometimes
d. Almost always
e. Always
f. I don't know / I prefer not to answer

b. Think about this NEW sentence:

"WHEN I STARTED I WAS NOT ABLE TO STOP".

(Same options that above)

c. Think about this NEW sentence:

"I FELT I NEEDED TO DO IT IN ORDER TO PERFORM"

(Same options that above)

d. Think about this NEW sentence:

"I CONTINUED DOING IT, EVEN IT GOT ME IN TROUBLE".

(Same options that above)

**ABOUT INYECTING STEROIDS AND OTHER SUBSTANCES**

Have you ever injected yourself (or been injected) anabolizing STEROIDS, such as testosterone or similar? [Answering is mandatory]

1. Yes
2. No *[skip 3 questions]*

And when did you do it for last time?

1. Last month
2. In last 6 months
3. In last 12 months
4. In last 5 years
5. More than 5 years ago

How old were you the first time you did it?

/__/__/

As SHARING we want to say using a syringe already used by another person, taking substances dissolved in a syringe used by another person or from the recipient where another person had introduced their syringe.

Considering this, have you ever SHARED when injecting steroids?

1. Yes

2. No

Have you ever injected yourself (or been injected) any DRUG FOR GETTING HIGH? It does not matter if it was related with sex or not.

1. Yes
2. No *[skip next section]*

And when did you do it for last time?

1. Last month
2. In last 6 months
3. In last 12 months
4. In last 5 years
5. More than 5 years ago

How old were you the first time you did it?

/__/__/

It was you who injected yourself, or another person?

1. I, myself *[skip 1 question]*
2. Another person

Who was that person?

1. A current sexual partner
2. A sporadic sexual partner
3. Your brother
4. Other relative
5. A very close friend
6. A drug dealer
7. An almost unknown person
8. Other people. Specify:

And which substance did you inject or be injected for the first time?

Transfer responses of those drugs consumed in any circumstance

1. Cocaine (powder or crack)

2. Heroine or opioids
3. Amphetamine/speed
4. Metamphetamine
5. Ecstasy
6. Mephedrone or similars
7. Ketamine

And that time you injected, did you do it in the previous 6 hours or during anal sex?

1. Yes
2. No

AND IN ALL YOUR LIFE, how many times have you injected yourself (or being injected) any substance in order to get high?

1. One
2. Less than 5
3. Less than 10
4. Less than 20
5. Less than 50
6. Less than 100
7. More than 100

Among the following drugs, which of them have you injected (or been injected) any time in your life? [multiple answer] Transfer responses of those drugs consumed in any circumstance

1. Cocaine (powder or crack)
2. Heroine or opioids
3. Amphetamine/speed
4. Metamphetamine
5. Écstasy
6. Mephedrone or similars
7. Ketamine

Now, tell us the three you have injected THE MOST. From more to less frequentyl

Write “1” in the first one, “2” in the second one, and “3” in the third.

(Transfer answers from last question)

And, have you injected drugs to get high in the previous 6 hours or during anal sex?

1. Yes
2. No *[skip 1 question]*

Which is the substance you have injected more frequently for anal sex?

As SHARING we want to say using a syringe already used by another person, taking substances dissolved in a syringe used by another person or from the recipient where another person had introduced their syringe.

Considering this, have you ever SHARED when injecting drugs to get high?

1. Yes
2. No *[skip 3 questions]*

When did you do it for the last time?

1. Last month
2. In the last 6 months
3. In the last 12 months
4. In the last 5 years
5. More than 5 years ago

And during ALL YOUR LIFE,

With how many different people do you think you have SHARED when injecting drugs to get high?

1. One
2. Less than 5
3. Less than 10
4. Less than 20
5. Less than 50
6. More than 50

And, among them, with how many the goal was to enhance sexual relations?

1. None
2. Less than a half
3. A half

4. More than a half
5. All

STILL SOMETHING MORE ABOUT YOU

How many inhabitants live in the place you live?

1. More than a million
2. More than 500,000
3. More than 100,000
4. More than 50,000
5. More than 10,000
6. Less than de 10,000

Your economic status is:

1. Very easy
2. Easy, it is fine
3. I have to limit some expenses in order to finish the month
4. I finish the month with economic difficulties
5. I finish the month with debts

FILTER: THOSE WHO WERE NOT BORN IN SPAIN BUT LIVE IN SPAIN, answer this question:

¿How long have you been living in Spain? (2 digits)

/__/__/ ***Write "0" years if you have been living for less than 1 year***

ABOUT HIV AUTO-TEST

We are going to talk about HIV autotest, we want to say: when people themselves want to know if they are infected by HIV, employ a kit to obtain a sample, and have the result in 15-20 minutes, without any professional support.

Do you know that in SPAIN, for more than one year ago, you can obtain a HIV auto-test at pharmacies without any medical recipe?

1. No, I did not know it [Skip next section]
2. I have heard about it, but I was not sure

3. Yes, I was informed

How did you get informed?

1. By a sexual partner
2. By friends
3. By a LGTB association
4. By a HIV/AIDS association
5. In a gay dating website
6. In media adressed to the gay community
7. In general media
8. In a website related to HIV
9. I saw it at a pharmacy
10. I saw a pharmaceutical advertisement in internet
11. I saw it in a vending machine
12. Other way, specify

FILTER: only continue those who said they knew it

For those who have heard about it, but were not sure, (answer 2) → to PREP

Have you ever done an HIV AUTO-TEST?

1. Yes
2. No [Skip 5 questions]

How many times?

1. One
2. Two
3. Three or more

When did you do it for the last time?

1. Less than 3 months ago
2. From 3 to 6 months ago
3. From 6 months to 1 year ago
4. From 1 to 2 years ago
5. From 2 to 5 years ago

6. More than 5 years ago

¿How did you obtain the LAST AUTO-TEST?

1. I bought it at a pharmacy in Spain
2. I bought it at an online pharmacy in Spain
3. I bought it in a vending machine in Spain
4. I bought it in a country where it is sold legally (by internet, at a pharmacy, etc.)
5. I bought it by internet without knowing if it was legally or not
6. Another way. Specify:

Were you alone or with another person when you did it?

1. Alone [Skip 1 question]
2. With another person

Who was with you?

1. My current partner
2. A sporadic partner
3. A friend
4. A relative
5. A NGO professional
6. A health professional
7. Another person, specify:

Have you ever considered seriously employing an auto-test instead of being tested at a health center?

1. Yes
2. No [Skip 1 question]

Which was the main reason for not using an auto-test at the end?

1. Taking the sample and interpreting the results must be done by a professional
2. A professional must be present to assess the person and explain the results
3. I am not sure about the test's quality, I have doubts about the fiability of the results
4. I do not want to pay 25€, I can be tested for HIV at other places without paying
5. Another reason. Specify

ABOUT PREP

SECTION FOR HIV-NEGATIVES (not to display this consideration in the questionnaire)

The PrEP is a tool for HIV-negative people consisting on taking a retroviral pill which reduces their risk of acquiring the infection.

Have you ever had PREP?

1. Yes
2. No [Skip until the end of the questionnaire]

When have you had it?

1. When I have needed it [Skip 3 questions]
2. Sometimes when I have needed it, and daily for certain period of time
3. I used to have it daily, but I do not do it any more
4. I have it daily [Skip 1 question]

For how much time have you had it daily?

1. For less than one month
2. For less than 3 months
3. For less than 6 months
4. For less than one year
5. For more than one year

[Skip 1 question]

How relevant the fact of having sex under the effect of drugs and without preservative has been a reason leading you to have PrEP?

1. Very relevant
2. Quite relevant
3. I am not sure
4. Not very important
5. Not important at all

[Skip until the end of the questionnaire]

If answer is 1 or 2 :

What proportion of all times you have had PREP it has been motivated for participating in sex sessions under the effect of drugs and without preservative?

1. Always
2. Most of them
3. Half of them
4. Less than a half
5. Never

[Skip until the end of the questionnaire]

ABOUT PREP

SECTION FOR HIV-POSITIVES (not to display this consideration in the questionnaire)

The PrEP is a tool for HIV-negative people consisting on taking a retroviral pill which reduces their risk of acquiring the infection.

Have you ever had PREP?

1. Yes
2. No [Skip until the end of the questionnaire]

When have you had it?

1. When I have needed it, never daily [Skip 2 questions]
2. Sometimes when I have needed it, and daily for certain period of time
3. Only daily

For how much time have you had it daily?

1. For less than one month
2. For less than 3 months
3. For less than 6 months
4. For less than one year
5. For more than one year

How relevant the fact of having sex under the effect of drugs and without preservative has been a reason leading you to have PrEP?

1. Very relevant
2. Quite relevant
3. I am not sure
4. Not very important
5. Not important at all

If answer is 1 or 2 :

How relevant the fact of having sex under the effect of drugs and without preservative has been a reason leading you to have PrEP?

1. Very relevant
2. Quite relevant
3. I am not sure
4. Not very important
5. Not important at all

THANK YOU VERY MUCH for your collaboration

We want to know the opinion of the highest number of MSM as possible. For so, please copy the link below and send it by mail, Whatsapp, Instagram or other ways to all your friends. Encourage them to participate.

https://es.surveymonkey.com/r/online_rds
