# Supplementary material for: Excessive Drinking Among Men Who Have Sex With Men Recruited From Web-Based Resources: Cross-sectional Questionnaire Study
Source: JMIR Public Health Surveill. 2022 Oct 31;8(10):e32888. doi: 10.2196/32888 (PMC9664322; doi:10.2196/32888)
Supplement: Multimedia Appendix 2 [file publichealth_v8i10e32888_app2.pdf]

SECRETARÍA DE ESTADO DE SERVICIOS SOCIALES E IGUALDAD

GOBIERNO DE ESPAÑA

MINISTERIO DE SANIDAD, SERVICIOS SOCIALES E IGUALDAD

DELEGACIÓN DEL GOBIERNO PARA EL PLAN NACIONAL SOBRE DROGAS

edADes

ENCUESTA SOBRE ALCOHOL Y DROGAS EN ESPAÑA 2017

CUESTIONARIO AUTORRELLENABLE

Nº DE RUTA					
PROVINCIA					
MUNICIPIO					
DISTRITO					
SECCIÓN					
Nº DE HOGAR EN LA RUTA					
Nº DE ENTREVISTADOR/A					
Nº DE CUESTIONARIO					

GRUPO ANÁLISIS E INVESTIGACIÓN

INSTRUCCIONES PARA EL ENTREVISTADO/A

En algunas preguntas se indaga sobre el **número de días** que usted ha consumido una determinada sustancia. Para orientarse sobre cómo debe responder a este tipo de preguntas revise, por favor, el siguiente ejemplo:

¿Cuántos días tomó usted una o más bebidas alcohólicas durante los ÚLTIMOS 12 MESES?

Primero lea, una a una, todas las categorías de las respuestas encerradas en la tabla situada abajo. Puede que necesite hacer un pequeño esfuerzo de memoria y realizar un cálculo aproximado para seleccionar la categoría que le corresponde.

Recuerde que 12 meses=52 semanas=365 días

ENTRE 1 – 3 DÍAS		1	ENTRE 30 – 150 DÍAS		5
ENTRE 4 – 9 DÍAS		2	MAS DE 150 DÍAS		6
ENTRE 10 – 19 DÍAS		3	NO HE TOMADO BEBIDAS ALCOHÓLICAS DURANTE LOS ÚLTIMOS 12 MESES		7
ENTRE 20 – 29 DÍAS		4	NUNCA HE TOMADO BEBIDAS ALCOHÓLICAS		8

Lea atentamente, y si lo necesita consulte con el entrevistador/a.

- Usted deberá marcar, tal como aparece en la tabla de más abajo, **la categoría "Entre 20-29 días" si en los ÚLTIMOS 12 MESES** sólo ha bebido en las celebraciones familiares tales como bautizos, bodas y cumpleaños. Esto podría equivaler a una media de 2 días al mes durante todo el año (12 meses) = **24 días.**

ENTRE 1 – 3 DÍAS		1	ENTRE 30 – 150 DÍAS		5
ENTRE 4 – 9 DÍAS		2	MAS DE 150 DÍAS		6
ENTRE 10 – 19 DÍAS		3	NO HE TOMADO BEBIDAS ALCOHÓLICAS DURANTE LOS ÚLTIMOS 12 MESES		7
ENTRE 20 – 29 DÍAS	X	4	NUNCA HE TOMADO BEBIDAS ALCOHÓLICAS		8

- Si el consumo que ha realizado en **LOS ÚLTIMOS 12 MESES** se limita al fin de semana (entendiendo como tal viernes, sábados y domingos) = 3 días x 52 semanas = **156 días.** En este caso deberá marcar **la categoría "Más de 150 días".**

ENTRE 1 – 3 DÍAS		1	ENTRE 30 – 150 DÍAS		5
ENTRE 4 – 9 DÍAS		2	MAS DE 150 DÍAS	X	6
ENTRE 10 – 19 DÍAS		3	NO HE TOMADO BEBIDAS ALCOHÓLICAS DURANTE LOS ÚLTIMOS 12 MESES		7
ENTRE 20 – 29 DÍAS		4	NUNCA HE TOMADO BEBIDAS ALCOHÓLICAS		8

También puede darse el caso de que su consumo varíe en determinadas épocas del año:

- **<u>En verano</u>**: si durante el año realiza un consumo de fin de semana, pero los meses de verano (junio, julio y agosto) además bebe 3 días entre semana:

  → Verano: 2 días del fin de semana + 3 días entre semana x 12 semanas (teniendo en cuenta que cada mes tiene 4 semanas) = 5 días x 12 semanas = **60 días.**

  → Resto del año: 2 días (sábado y domingo) x 40 semanas = 2 x 40 = **80 días.**

  En total **140 días.**

- **<u>En Navidad</u>**: si durante la semana de Navidad el consumo lo hace diario, manteniendo el resto del año un consumo de fin de semana:

  → Navidad: 7 días x 2 semanas = **14 días.**

  → Resto del año: 2 días (sábado y domingo) x 50 semanas = 2 x 50 = **100 días.**

  En total **114 días.**

Hay preguntas, como la del ejemplo anterior, que hacen referencia a un periodo concreto. Es muy importante que **esté atento e identifique los distintos periodos** para poder contestar correctamente:

– EN TODA LA VIDA
– ÚLTIMOS 12 MESES
– ÚLTIMOS 30 DÍAS

Si se ha equivocado y quiere corregir una respuesta que ya ha marcado, rodee con un círculo completamente la respuesta incorrecta (X) y marque con una **x** la respuesta correcta.

¿Cuántos días tomó Vd. una o más bebidas alcohólicas durante los ÚLTIMOS 12 MESES?

Respuesta incorrecta →

ENTRE 1– 3 DÍAS	(X)	1	ENTRE 30 – 150 DÍAS		5
ENTRE 4 – 9 DÍAS		2	MAS DE 150 DÍAS		6
ENTRE 10 – 19 DÍAS		3	NO HE TOMADO BEBIDAS ALCOHÓLICAS DURANTE LOS ÚLTIMOS 12 MESES		7
ENTRE 20 – 29 DÍAS	X	4	NUNCA HE TOMADO BEBIDAS ALCOHÓLICAS		8

Respuesta correcta →

Si no entiende bien las instrucciones, por favor coméntelo con el entrevistador/a.

A L C O H O L

Marque con una "X" la casilla (☐) correspondiente a su respuesta
Recuerde que **sólo ha de marcar una casilla** en cada pregunta

SE CONSIDERA BEBIDA ALCOHÓLICA CUALQUIER TIPO DE SUSTANCIA QUE CONTENGA ALCOHOL, ENTRE LAS QUE SE INCLUYEN:

- **VINO, CHAMPÁN, CAVA**
- **CERVEZA, SIDRA** (cañas, claras…)
- **VERMÚ, JEREZ, FINOS**
- **COMBINADOS o CUBATAS** (copas de bebidas alcohólicas de alta graduación mezcladas con refrescos, zumos,… Se incluyen los mojitos)
- **LICORES DE FRUTAS** (pacharán, licores de manzana, pera, melocotón, melón...)
- **LICORES FUERTES o BEBIDAS DESTILADAS** (coñac, brandy, ginebra, ron, whisky, crema de orujo, crema de ron, tequila, carajillo…)

A1. ¿Ha tomado usted, ALGUNA VEZ, aunque fuera sólo una vez, cualquier clase de bebida alcohólica?

SÍ, ALGUNA VEZ HE TOMADO UNA BEBIDA ALCOHÓLICA	☐	1
NUNCA HE TOMADO UNA BEBIDA ALCOHÓLICA	☐	2

A2. ¿Qué EDAD tenía la PRIMERA VEZ que tomó cualquier clase de bebida alcohólica? No incluya sorbos de la bebida de otra persona.
Si Vd. no puede recordar exactamente qué edad tenía, indique una edad aproximada. Ponga los años que tenía sobre la línea o marque con una "X" la casilla.

ANOTE EDAD DEL PRIMER CONSUMO DE CUALQUIER BEBIDA ALCOHÓLICA:________AÑOS

NUNCA HE TOMADO UNA BEBIDA ALCOHÓLICA	☐	1

A3. Ahora piense en los ÚLTIMOS 12 MESES. ¿Cuántos días tomó Vd. una o más bebidas alcohólicas durante los ÚLTIMOS 12 MESES?
Recuerde el ejemplo que leyó al inicio del cuestionario.

ENTRE 1 – 3 DÍAS	☐	1	ENTRE 30 – 150 DÍAS	☐	5
ENTRE 4 – 9 DÍAS	☐	2	MAS DE 150 DÍAS	☐	6
ENTRE 10 – 19 DÍAS	☐	3	NO HE TOMADO BEBIDAS ALCOHÓLICAS DURANTE LOS ÚLTIMOS 12 MESES	☐	7
ENTRE 20 – 29 DÍAS	☐	4	NUNCA HE TOMADO BEBIDAS ALCOHÓLICAS	☐	8

A4. Durante estos ÚLTIMOS 12 MESES, ¿cuántos días se emborrachó?

ENTRE 1– 3 DÍAS	☐	1	NO HE TOMADO BEBIDAS ALCOHÓLICAS DURANTE LOS ÚLTIMOS 12 MESES	☐	6
ENTRE 4 – 9 DÍAS	☐	2	NUNCA HE TOMADO BEBIDAS ALCOHÓLICAS	☐	7
ENTRE 10 – 19 DÍAS	☐	3	NO ME HE EMBORRACHADO EN LOS ÚLTIMOS 12 MESES	☐	8
ENTRE 20 – 29 DÍAS	☐	4	NUNCA ME HE EMBORRACHADO	☐	9
30 DÍAS ó MÁS	☐	5			

A5. En los ÚLTIMOS 12 MESES, ¿Cuántos días ha bebido lo suficiente para sentir sensación de inestabilidad o dificultad para hablar?

ENTRE 1 – 3 DÍAS	☐ 1	NO HE TOMADO BEBIDAS ALCOHÓLICAS DURANTE LOS ÚLTIMOS 12 MESES	☐ 6
ENTRE 4 – 9 DÍAS	☐ 2	NUNCA HE TOMADO BEBIDAS ALCOHÓLICAS	☐ 7
ENTRE 10 – 19 DÍAS	☐ 3	NO HE TENIDO TRAS BEBER SENSACIÓN DE INESTABILIDAD O DIFICULTAD PARA HABLAR EN LOS ÚLTIMOS 12 MESES	☐ 8
ENTRE 20 – 29 DÍAS	☐ 4	NUNCA HE TENIDO TRAS BEBER SENSACIÓN DE INESTABILIDAD O DIFICULTAD PARA HABLAR	☐ 9
30 DÍAS ó MÁS	☐ 5		

A6. ¿En los últimos 12 meses, con qué frecuencia ha hecho usted "botellón"?

2 ó MÁS DÍAS A LA SEMANA	☐ 1
3 ó 4 DÍAS AL MES	☐ 2
1 ó 2 DÍAS AL MES	☐ 3
MENOS DE 1 DÍA AL MES (ENTRE 4 y 11 DÍAS AL AÑO)	☐ 4
DE 1 a 3 DÍAS AL AÑO	☐ 5
NO HE HECHO BOTELLÓN EN EL ÚLTIMO AÑO	☐ 6
NUNCA HE HECHO BOTELLÓN	☐ 7

A7. Las personas beben alcohol por distintos motivos. Nos gustaría saber cuáles son para usted los motivos principales para beber. Si piensa en las ocasiones en que ha bebido en los ÚLTIMOS 12 MESES, ya sea cerveza, vino, combinados o licores, ¿Con qué frecuencia ha bebido...

	NUNCA	ALGUNA VEZ	LA MITAD DE LAS VECES	CASI SIEMPRE	SIEMPRE
PORQUE **LE GUSTA CÓMO SE SIENTE** DESPUÉS DE BEBER?	☐ 1	☐ 2	☐ 3	☐ 4	☐ 5
PORQUE ES **DIVERTIDO O** PORQUE **ANIMA LAS FIESTAS** Y CELEBRACIONES?	☐ 1	☐ 2	☐ 3	☐ 4	☐ 5
SÓLO **PARA EMBORRACHARSE**?	☐ 1	☐ 2	☐ 3	☐ 4	☐ 5
PARA **ENCAJAR EN UN GRUPO** QUE LE GUSTA O PARA **NO SENTIRSE EXCLUIDO** (EN RELACIÓN CON LOS DEMÁS)?	☐ 1	☐ 2	☐ 3	☐ 4	☐ 5
PORQUE LE **AYUDA CUANDO SE SIENTE DEPRIMIDO** O CUANDO NECESITA **OLVIDARSE** DE TODO?	☐ 1	☐ 2	☐ 3	☐ 4	☐ 5
PORQUE CREE QUE ES **SALUDABLE O** PORQUE FORMA PARTE DE UNA **ALIMENTACIÓN EQUILIBRADA**?	☐ 1	☐ 2	☐ 3	☐ 4	☐ 5

NO HE TOMADO UNA BEBIDA ALCOHÓLICA EN LOS ÚLTIMOS 12 MESES	☐ 9
NUNCA HE TOMADO UNA BEBIDA ALCOHÓLICA	☐ 0

Las siguientes preguntas se refieren solamente a los ÚLTIMOS 30 DÍAS

A8. Centrándose en los ÚLTIMOS 30 DÍAS, ¿cuántos días tomó Vd. una o más bebidas alcohólicas?

ENTRE 1 – 3 DÍAS	☐ 1	30 DÍAS	☐ 5
ENTRE 4 – 9 DÍAS	☐ 2	NO HE TOMADO BEBIDAS ALCOHÓLICAS DURANTE LOS ÚLTIMOS 30 DÍAS	☐ 6
ENTRE 10 – 19 DÍAS	☐ 3	NUNCA HE TOMADO BEBIDAS ALCOHÓLICAS	☐ 7
ENTRE 20 – 29 DÍAS	☐ 4		

A9. Durante los ÚLTIMOS 30 DÍAS, ¿cuántos días se emborrachó?

ENTRE 1 – 3 DÍAS	☐ 1	NO HE TOMADO BEBIDAS ALCOHÓLICAS DURANTE LOS ÚLTIMOS 30 DÍAS	☐ 6
ENTRE 4 – 9 DÍAS	☐ 2	NUNCA HE TOMADO BEBIDAS ALCOHÓLICAS	☐ 7
ENTRE 10 – 19 DÍAS	☐ 3	NO ME HE EMBORRACHADO EN LOS ÚLTIMOS 30 DÍAS	☐ 8
ENTRE 20 – 29 DÍAS	☐ 4	NUNCA ME HE EMBORRACHADO	☐ 9
30 DÍAS	☐ 5		

Durante los ÚLTIMOS 30 DÍAS, ¿cuántos días ha tomado en la misma ocasión 5 ó más bebidas alcohólicas (si usted es hombre) o 4 ó más bebidas alcohólicas (si usted es mujer)? Entendiendo por "ocasión" cuando se toman varias bebidas seguidas o en un plazo de un par de horas. No excluya las bebidas tomadas durante las comidas. Ponga sobre la línea el número de días.

A10. HOMBRE:

ANOTE EL **Nº DE DÍAS** EN QUE HA TOMADO 5 ó MÁS BEBIDAS EN LA MISMA OCASIÓN: ______ días

NO HE TOMADO 5 ó MÁS BEBIDAS ALCOHÓLICAS EN LA MISMA OCASIÓN DURANTE LOS ÚLTIMOS 30 DÍAS	☐ 1
NUNCA HE TOMADO UNA BEBIDA ALCOHÓLICA	☐ 2
NO HE TOMADO BEBIDAS ALCOHÓLICAS DURANTE LOS ÚLTIMOS 30 DÍAS	☐ 3

A11. MUJER:

ANOTE EL **Nº DE DÍAS** EN QUE HA TOMADO 4 ó MÁS BEBIDAS EN LA MISMA OCASIÓN: ______ días

NO HE TOMADO 4 ó MÁS BEBIDAS ALCOHÓLICAS EN LA MISMA OCASIÓN DURANTE LOS ÚLTIMOS 30 DÍAS	☐ 1
NUNCA HE TOMADO UNA BEBIDA ALCOHÓLICA	☐ 2
NO HE TOMADO BEBIDAS ALCOHÓLICAS DURANTE LOS ÚLTIMOS 30 DÍAS	☐ 3

Las siguientes preguntas se refieren solamente a los ÚLTIMOS 7 DÍAS

A12. En los ÚLTIMOS 7 DÍAS, ¿Cuántos días ha consumido usted alguna bebida alcohólica?

Marque de 0 a 7 el número de días que ha consumido bebidas alcohólicas en los últimos 7 días siendo **0 días ningún día** de los últimos 7 días y **7 días todos los días** durante los últimos 7 días.

	**0 días**	**1 día**	**2 días**	**3 días**	**4 días**	**5 días**	**6 días**	**7 días**
NÚMERO DE **DÍAS QUE HA CONSUMIDO ALGUNA BEBIDA ALCOHÓLICA** EN LOS ÚLTIMOS 7 DÍAS	☐ 0	☐ 1	☐ 2	☐ 3	☐ 4	☐ 5	☐ 6	☐ 7

A13. En los ÚLTIMOS 7 DÍAS ¿Cuántos vasos o copas de las siguientes bebidas alcohólicas ha consumido en total? Anote el número de vasos o copas de estas bebidas que ha consumido en total en los últimos 7 días, si no ha consumido en los últimos 7 días marque 0.

		**Nº total de bebidas alcohólicas consumidas EN LOS ÚLTIMOS 7 DÍAS**
Nº de vasos o copas de **VINO/CHAMPÁN o CAVA**	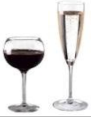	|__|__|
Nº de vasos, cañas o quintos de **CERVEZA o SIDRA**	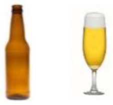	|__|__|
Nº de vasos o copas de **VERMÚ, JEREZ o FINO**	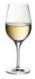	|__|__|
Nº de vasos de **COMBINADOS o CUBATAS**	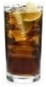	|__|__|
Nº de copas de **LICORES DE FRUTAS SOLOS (PACHARÁN, MANZANA)**	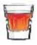	|__|__|
Nº de copas de **LICORES FUERTES SOLOS (COÑAC, GINEBRA, WHISKY)**	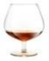	|__|__|

NO HE TOMADO BEBIDAS ALCOHÓLICAS DURANTE LOS ÚLTIMOS 7 DÍAS	☐ 1
NUNCA HE TOMADO UNA BEBIDA ALCOHÓLICA EN MI VIDA	☐ 2

Las siguientes preguntas se refieren al consumo de bebidas energéticas

A14. ¿Ha tomado en los ÚLTIMOS 30 DÍAS bebidas energéticas (Red Bull, Burn, Monster…)? Conteste "No" si lo único que hizo fue dar un sorbo o probar de la bebida de otra persona.

SÍ	☐ 1
NO	☐ 2

A15. ¿Ha tomado en los ÚLTIMOS 30 DÍAS bebidas energéticas (Red Bull, Burn, Monster…) mezcladas con bebidas alcohólicas? Conteste "No" si lo único que hizo fue dar un sorbo o probar de la bebida de otra persona.

SÍ	☐ 1
NO	☐ 2

Las siguientes preguntas se refieren al consumo de bebidas alcohólicas en general

A16. Conteste a esta pregunta SÓLO SI ES USTED UNA MUJER. Si ha estado usted EMBARAZADA o está actualmente embarazada, ¿Ha consumido o consume cualquier bebida alcohólica durante el embarazo?

SÍ	☐ 1
NO	☐ 2
No he estado embarazada NUNCA	☐ 9

A17. A continuación le indicamos algunas afirmaciones sobre actitudes ante las POLÍTICAS EN MATERIA DE ALCOHOL. Para cada afirmación, indique si está de acuerdo o en desacuerdo:

	De acuerdo	En desacuerdo
El alcohol es un producto como cualquier otro y no necesita **ninguna restricción** especial	☐1	☐2
Las **personas adultas** son lo suficientemente responsables para **protegerse a sí mismas** de los daños relacionados con el consumo de alcohol	☐1	☐2
Los **poderes públicos** tienen la responsabilidad de **proteger a las personas** de los daños relacionados con su consumo de alcohol	☐1	☐2
La **educación y la información** en materia de alcohol deberían ser las políticas más importantes para reducir los daños relacionados con el alcohol	☐1	☐2
Se debería **prohibir la publicidad** de bebidas alcohólicas	☐1	☐2
En los envases de las bebidas alcohólicas se deberían mostrar **advertencias sobre los daños** relacionados con el alcohol	☐1	☐2
Los **padres o tutores legales**, y no las autoridades legales, deben decidir a qué **edad se permite a los hijos consumir** bebidas alcohólicas	☐1	☐2
El **patrocinio de deportistas**, equipos deportivos o eventos deportivos por parte de la industria del alcohol debieran estar prohibidos por ley	☐1	☐2

Ahora vamos a volver a preguntarle algunas cosas sobre el **CONSUMO DE BEBIDAS ALCOHÓLICAS EN LOS 12 ÚLTIMOS MESES.**

Muy importante:

- SI NO HA CONSUMIDO ALCOHOL EN LOS ÚLTIMOS 12 MESES, no conteste este bloque y pase a la pregunta T1 (Tabaco)

- SI HA CONSUMIDO ALCOHOL EN LOS ÚLTIMOS 12 MESES, DEBE CONTESTAR TODAS LAS PREGUNTAS porque forman parte de un cuestionario y deben analizarse conjuntamente.

AU1. ¿Con qué frecuencia consume bebidas alcohólicas?

Nunca	☐	1
Una o menos veces al mes	☐	2
2 a 4 veces al mes	☐	3
2 ó 3 veces a la semana	☐	4
4 o más veces a la semana	☐	5

AU2. ¿Cuántas bebidas alcohólicas consume normalmente cuando bebe?

1 ó 2	☐	1
3 ó 4	☐	2
5 ó 6	☐	3
7 a 9	☐	4
10 o más	☐	5

AU3. ¿Con qué frecuencia se toma 6 o más bebidas alcohólicas en un solo día?

Nunca	☐	1
Menos de una vez / mes	☐	2
Mensualmente	☐	3
Semanalmente	☐	4
A diario o casi a diario	☐	5

AU4. ¿Con qué frecuencia, en el curso del último año, ha sido incapaz de parar de beber una vez que había empezado?

Nunca	☐	1
Menos de una vez / mes	☐	2
Mensualmente	☐	3
Semanalmente	☐	4
A diario o casi a diario	☐	5

AU5. ¿Con qué frecuencia, en el curso del último año, no pudo atender sus obligaciones porque había bebido?

Nunca	☐	1
Menos de una vez / mes	☐	2
Mensualmente	☐	3
Semanalmente	☐	4
A diario o casi a diario	☐	5

AU6. ¿Con qué frecuencia, en el curso del último año, ha necesitado beber en ayunas para recuperarse después de haber bebido mucho el día anterior?

Nunca	☐	1
Menos de una vez / mes	☐	2
Mensualmente	☐	3
Semanalmente	☐	4
A diario o casi a diario	☐	5

AU7. ¿Con qué frecuencia, en el curso del último año, ha tenido remordimientos o sentimientos de culpa después de haber bebido?

Nunca	☐	1
Menos de una vez / mes	☐	2
Mensualmente	☐	3
Semanalmente	☐	4
A diario o casi a diario	☐	5

AU8. ¿Con qué frecuencia, en el curso del último año, no ha podido recordar lo que sucedió la noche anterior porque había estado bebiendo?

Nunca	☐	1
Menos de una vez / mes	☐	2
Mensualmente	☐	3
Semanalmente	☐	4
A diario o casi a diario	☐	5

AU9. Usted, o alguna otra persona, ¿ha resultado herida porque usted había bebido?

No	☐	1
Sí, pero no en el curso del último año	☐	2
Sí, en el último año	☐	3

AU10. ¿Algún familiar, amigo, médico o profesional sanitario ha mostrado preocupación por su consumo de alcohol, o le ha sugerido que deje de beber?

No	☐	1
Sí, pero no en el curso del último año	☐	2
Sí, en el último año	☐	3

VD. HA TERMINADO LAS PREGUNTAS ACERCA DEL ALCOHOL. GRACIAS.
POR FAVOR, AHORA CONTINUE LEYENDO Y RESPONDIENDO POR VD. MISMO LAS PREGUNTAS QUE SIGUEN Y SI TIENE ALGUNA DUDA PUEDE CONSULTAR AL ENTREVISTADOR/A EN CUALQUIER MOMENTO

TABACO

Marque con una "X" la casilla (☐) correspondiente a su respuesta
Recuerde que **sólo ha de marcar una casilla** en cada pregunta

T1. Indique si usted ha consumido UN CIGARRILLO U OTRO TIPO DE TABACO, aunque fuera sólo una o dos caladas ALGUNA VEZ EN LA VIDA, y/o en los ÚLTIMOS 12 MESES, y/o en los ÚLTIMOS 30 DÍAS y/o A DIARIO EN LOS ÚLTIMOS 30 DÍAS. Si ha consumido anote en el recuadro correspondiente la EDAD en que lo consumió por PRIMERA VEZ.

	¿Ha consumido ALGUNA VEZ EN LA VIDA?		¿Ha consumido alguna vez en los ÚLTIMOS 12 MESES?		¿Ha consumido alguna vez en los ÚLTIMOS 30 DÍAS?		¿Ha consumido alguna vez A DIARIO en los ÚLTIMOS 30 DÍAS?		EDAD en que consumió por primera vez en su vida
	No	Sí	No	Sí	No	Sí	No	Sí	
**CIGARRILLO U OTRO TIPO DE TABACO, aunque fuera sólo una o dos caladas**	☐1	☐2	☐1	☐2	☐1	☐2	☐1	☐2	

T2. ¿Cuándo ha sido la PRIMERA VEZ que ha fumado, un cigarrillo u otro tipo de tabaco?

HACE UN AÑO O MENOS	☐	1
HACE MÁS DE 1 AÑO	☐	2
NUNCA HE FUMADO UN CIGARRILLO U OTRO TIPO DE TABACO	☐	3

T3. Durante los ÚLTIMOS 30 DÍAS, por término medio, ¿qué número de cigarrillos o puros solía fumar cada día que fumaba? Anote el número medio o marque con una "X" el recuadro correspondiente.

CIGARRILLOS	PUROS
ANOTE EL Nº:________	ANOTE EL Nº:________

NO HE FUMADO UN CIGARRILLO o PURO EN LOS ÚLTIMOS 30 DÍAS	☐	1
NUNCA HE FUMADO UN CIGARRILLO O PURO	☐	2

T4. Durante los ÚLTIMOS 30 DÍAS, si ha fumado cigarrillos, ¿qué tipo de cigarrillos ha fumado?

CIGARRILLOS DE CAJETILLA	☐	1
TABACO DE LIAR	☐	2
DE LOS 2 TIPOS	☐	3
NO HE FUMADO EN LOS ÚLTIMOS 30 DÍAS	☐	4
NUNCA HE FUMADO EN MI VIDA	☐	5

T5. ¿Ha habido, alguna vez, un período en su vida en que Vd. haya fumado tabaco TODOS LOS DÍAS?

SÍ, ALGUNA VEZ HE FUMADO TODOS LOS DÍAS	☐	1
NO HE FUMADO EN NINGUNA OCASIÓN TODOS LOS DÍAS	☐	2
NUNCA HE FUMADO UN CIGARRILLO U OTRO TIPO DE TABACO	☐	3

T6. ¿Qué edad tenía cuando comenzó a fumar tabaco TODOS LOS DÍAS?
Ponga sobre la línea los años que tenía o marque el recuadro correspondiente.

ANOTE LA **EDAD:**_______AÑOS

NUNCA HE FUMADO TODOS LOS DÍAS	☐	1
NUNCA HE FUMADO EN MI VIDA	☐	2

T7. ¿Durante cuántos años fumó TODOS LOS DÍAS?
Ponga sobre la línea los años o marque el recuadro correspondiente.

ANOTE EL **Nº DE AÑOS**:_______ AÑOS

HE FUMADO TODOS LOS DÍAS, PERO DURANTE MENOS DE 1 AÑO COMPLETO	☐	1
HE FUMADO ALGUNA VEZ, PERO NUNCA TODOS LOS DÍAS	☐	2
NUNCA HE FUMADO EN MI VIDA	☐	3

T8. ¿Cuánto tiempo ha pasado desde la última vez que Vd. fumó un cigarrillo u otro tipo de tabaco?

MENOS DE 30 DÍAS	☐	1	MÁS DE 12 MESES	☐	3
MÁS DE UN MES PERO MENOS DE UN AÑO	☐	2	NUNCA HE FUMADO UN CIGARILLO U OTRO TIPO DE TABACO	☐	4

T9. ¿Se ha planteado alguna vez dejar de fumar?

SÍ Y LO HE INTENTADO	☐	1
SÍ, PERO NO LO HE INTENTADO	☐	2
NO ME LO HE PLANTEADO	☐	3
NO FUMO	☐	4

Las siguientes preguntas se refieren al consumo de cigarrillos electrónicos

CE1. Indique si usted ha consumido <u>CIGARRILLOS ELECTRÓNICOS (vapear)</u> ALGUNA VEZ EN LA VIDA, y/o en los ÚLTIMOS 12 MESES, y/o en los ÚLTIMOS 30 DÍAS y/o A DIARIO EN LOS ÚLTIMOS 30 DÍAS. Si ha consumido anote en el recuadro correspondiente la EDAD en que lo consumió por PRIMERA VEZ.

	¿Ha consumido ALGUNA VEZ EN LA VIDA?		¿Ha consumido alguna vez en los ÚLTIMOS 12 MESES?		¿Ha consumido alguna vez en los ÚLTIMOS 30 DÍAS?		¿Ha consumido alguna vez A DIARIO en los ÚLTIMOS 30 DÍAS?		EDAD en que consumió <u>por primera vez en su vida</u>
	No	Sí	No	Sí	No	Sí	No	Sí	
**<u>CIGARRILLOS ELECTRÓNICOS (vapear)</u>**	☐1	☐2	☐1	☐2	☐1	☐2	☐1	☐2	

CE2. Si ha fumado CIGARRILLOS ELECTRÓNICOS (vapear), ¿Qué tipo de cartucho o líquido emplea para recargar su cigarrillo electrónico?

CARTUCHOS O LÍQUIDOS PARA CIGARRILLOS ELECTRÓNICOS **CON** NICOTINA	☐	1
CARTUCHOS O LÍQUIDOS PARA CIGARRILLOS ELECTRÓNICOS **SIN** NICOTINA	☐	2
DE LOS 2 TIPOS	☐	3
NO HE FUMADO CIGARRILLOS ELECTRÓNICOS NUNCA	☐	4

CE3. Si ha fumado CIGARRILLOS ELECTRÓNICOS (vapear), ¿Utiliza los CIGARRILLOS ELECTRÓNICOS (vapear) como <u>sustituto del tabaco,</u> para <u>reducir su consumo</u> o <u>dejar de fumar</u>?

SÍ	☐	1
NO	☐	2
NO HE FUMADO CIGARRILLOS ELECTRÓNICOS NUNCA	☐	3

VD. HA TERMINADO LAS PREGUNTAS ACERCA DEL TABACO. GRACIAS.
POR FAVOR, AHORA CONTINUE LEYENDO Y RESPONDIENDO POR VD. MISMO LAS PREGUNTAS QUE SIGUEN Y SI TIENE ALGUNA DUDA PUEDE CONSULTAR AL ENTREVISTADOR/A EN CUALQUIER MOMENTO

TRANQUILIZANTES / SEDANTES
[medicamentos para calmar los nervios o la ansiedad]
Y SOMNÍFEROS
[medicamentos para dormir]

<u>INCLUYA</u>: Lexatín, orfidal, noctamid, trankimazin, rohipnol, tranxilium, diacepam, valium, stilnox, zolpidem, hipnóticos, benzos, benzodiazepinas, barbitúricos, etc.

<u>NO INCLUYA</u>: valeriana, pasiflora, dormidina, melatonina.

TS1. Indique si usted ha consumido <u>TRANQUILIZANTES, SEDANTES y/o SOMNIFEROS</u> (medicamentos para calmar los nervios o la ansiedad o medicamentos para dormir) ALGUNA VEZ EN LA VIDA, y/o en los ÚLTIMOS 12 MESES, y/o en los ÚLTIMOS 30 DÍAS y/o A DIARIO EN LOS ÚLTIMOS 30 DÍAS. Si ha consumido anote en el recuadro correspondiente la EDAD en que los consumió por PRIMERA VEZ.

	¿Ha consumido ALGUNA VEZ EN LA VIDA?		¿Ha consumido alguna vez en los ÚLTIMOS 12 MESES?		¿Ha consumido alguna vez en los ÚLTIMOS 30 DÍAS?		¿Ha consumido alguna vez A DIARIO en los ÚLTIMOS 30 DÍAS?		EDAD en que consumió <u>por primera vez en su vida</u>
	No	Sí	No	Sí	No	Sí	No	Sí	
**<u>TRANQUILIZANTES, SEDANTES y/o SOMNIFEROS</u>** (medicamentos para calmar los nervios o la ansiedad o medicamentos para dormir)	☐ 1	☐ 2	☐ 1	☐ 2	☐ 1	☐ 2	☐ 1	☐ 2	

TS2. ¿Cuándo ha sido la <u>PRIMERA VEZ</u> que ha consumido <u>TRANQUILIZANTES, SEDANTES y/o SOMNIFEROS</u> (medicamentos para calmar los nervios o la ansiedad o medicamentos para dormir)?

HACE UN AÑO O MENOS	☐	1
HACE MÁS DE 1 AÑO	☐	2
NUNCA HE CONSUMIDO TRANQUILIZANTES, SEDANTES y/o SOMNÍFEROS	☐	3

TRANQUILIZANTES/SEDANTES
SIN RECETA O PARA USO NO MÉDICO
[medicamentos para calmar los nervios o la ansiedad]
Y SOMNÍFEROS SIN RECETA O PARA USO NO MÉDICO
[medicamentos para dormir]

A continuación las siguientes preguntas se refieren a los mismos medicamentos cuando se han obtenido SIN RECETA MÉDICA O PARA USO NO MÉDICO es decir:

- **El médico se lo recetó a usted en alguna ocasión pero lo ha seguido tomando sin control por su médico, en mayor cantidad o durante más tiempo que lo que le indicó su médico.**
- **El médico se lo ha recetado a otra persona de la familia y Vd. los toma de vez en cuando**
- **Los ha conseguido por otros medios distintos a la receta médica**

Marque con una "X" la casilla (☐) correspondiente a su respuesta
Recuerde que **sólo ha de marcar una casilla** en cada pregunta.

TS3. Indique si usted ha consumido TRANQUILIZANTES, SEDANTES y/o SOMNIFEROS SIN RECETA O PARA USO NO MÉDICO (medicamentos para calmar los nervios o la ansiedad o medicamentos para dormir) ALGUNA VEZ EN LA VIDA, y/o en los ÚLTIMOS 12 MESES, y/o en los ÚLTIMOS 30 DÍAS y/o A DIARIO EN LOS ÚLTIMOS 30 DÍAS. Si ha consumido anote en el recuadro correspondiente la EDAD en que la consumió por PRIMERA VEZ.

	¿Ha consumido ALGUNA VEZ EN LA VIDA?		¿Ha consumido alguna vez en los ÚLTIMOS 12 MESES?		¿Ha consumido alguna vez en los ÚLTIMOS 30 DÍAS?		¿Ha consumido alguna vez A DIARIO en los ÚLTIMOS 30 DÍAS?		EDAD en que consumió por primera vez en su vida
	No	Sí	No	Sí	No	Sí	No	Sí	
**TRANQUILIZANTES, SEDANTES y/o SOMNIFEROS SIN RECETA O PARA USO NO MÉDICO** (medicamentos para calmar los nervios o la ansiedad o medicamentos para dormir)	☐1	☐2	☐1	☐2	☐1	☐2	☐1	☐2	

TS4. En los ÚLTIMOS 12 MESES, ¿Ha consumido TRANQUILIZANTES, SEDANTES y/o SOMNIFEROS SIN RECETA O PARA USO NO MÉDICO (medicamentos para calmar los nervios o la ansiedad o medicamentos para dormir) en la mismo ocasión junto con…?
Entendiendo por "ocasión" en un plazo de un par de horas

	**SÍ**	**NO**
CON **ALCOHOL**?	☐1	☐2
CON **DROGAS ILEGALES** (CANNABIS, COCAÍNA, HEROÍNA…)?	☐1	☐2
CON **ALCOHOL Y DROGAS ILEGALES** (CANNABIS, COCAÍNA, HEROÍNA…) A LA VEZ?	☐1	☐2

**NO** HE TOMADO TRANQUILIZANTES, SEDANTES y/o SOMNIFEROS SIN RECETA O PARA USO NO MÉDICO EN LOS **ÚLTIMOS 12 MESES**	☐ 9
**NUNCA** HE TOMADO TRANQUILIZANTES, SEDANTES y/o SOMNIFEROS SIN RECETA O PARA USO NO MÉDICO	☐ 0

TS5. Piense en la última vez que tomó TRANQUILIZANTES, SEDANTES y/o SOMNIFEROS SIN RECETA O PARA USO NO MÉDICO (medicamentos para calmar los nervios o la ansiedad o medicamentos para dormir): ¿Cuáles fueron las razones de su consumo?
Puede marcar varias opciones

PARA EXPERIMENTAR O PROBAR LO QUE **SE SENTÍA** TOMANDO ESE MEDICAMENTO	☐	1
PARA **COLOCARME**	☐	2
PARA **SOBRELLEVAR** LAS SITUACIONES HABITUALES DEL **DÍA A DÍA**	☐	3
PARA INCREMENTAR O DISMINUIR LOS **EFECTOS DE DROGAS** QUE HABÍA TOMADO	☐	4
PARA **AFRONTAR** ALGUNOS **PROBLEMAS** VITALES (DIVORCIO, MUERTE DE UN FAMILIAR, DESPIDOS…)	☐	5
POR SER VÍCTIMA DE **VIOLENCIA DE GÉNERO**	☐	6
NUNCA HE TOMADO TRANQUILIZANTES, SEDANTES y/o SOMNIFEROS SIN RECETA O PARA USO NO MÉDICO	☐	9

TS6. Piense en la última vez que tomó TRANQUILIZANTES, SEDANTES y/o SOMNIFEROS SIN RECETA O PARA USO NO MÉDICO (medicamentos para calmar los nervios o la ansiedad o medicamentos para dormir) y seleccione el medio por el que los consiguió. Marque sólo una opción.

A TRAVÉS DE UNA **RECETA MÉDICA** QUE ME RECETARON A MI	☐	1
A TRAVÉS DE UN **AMIGO O FAMILIAR**	☐	2
A TRAVÉS DE UN **TRAFICANTE** O A UNA **PERSONA DESCONOCIDA**	☐	3
A TRAVÉS DE **INTERNET**	☐	4
A TRAVÉS DE UNA **RECETA MÉDICA FALSA**	☐	5
LO COMPRÉ EN UNA **FARMACIA SIN RECETA**	☐	6
LO CONSEGUÍ **DE OTRA MANERA**	☐	7
NUNCA HE TOMADO TRANQUILIZANTES, SEDANTES y/o SOMNIFEROS SIN RECETA O PARA USO NO MÉDICO	☐	9

TS7. Estaría dispuesto a intentar conseguir ILEGALMENTE TRANQUILIZANTES, SEDANTES y/o SOMNIFEROS (medicamentos para calmar los nervios o la ansiedad o medicamentos para dormir) si no pudiera obtenerlos a través de receta médica?

SÍ	☐	1
NO	☐	2

**VD. HA TERMINADO LAS PREGUNTAS ACERCA DE TRANQUILIZANTES, SEDANTES y/o SOMNÍFEROS. GRACIAS.
POR FAVOR, AHORA CONTINUE LEYENDO Y RESPONDIENDO POR VD. MISMO LAS PREGUNTAS QUE SIGUEN Y SI TIENE ALGUNA DUDA PUEDE CONSULTAR AL ENTREVISTADOR/A EN CUALQUIER MOMENTO**

OTRAS SUSTANCIAS

CONTESTE AHORA, POR FAVOR, A ALGUNAS PREGUNTAS SOBRE OTROS TIPOS DE SUSTANCIAS:

- **CANNABIS, MARIHUANA o HACHÍS** [aceite de hachís, chocolate, porro, costo, hierba]
- **COCAÍNA EN POLVO** [cocaína, perico, farlopa, clorhidrato de cocaína]
- **COCAÍNA EN FORMA DE BASE** [base, cocaína cocinada, basuco, boliches, crack, roca]
- **ÉXTASIS U OTRAS DROGAS DE SÍNTESIS** [pastis, pirulas, cristal, MDMA]
- **ANFETAMINAS O SPEED** [anfetas]
- **ALUCINÓGENOS** [LSD, ácido, tripi, setas mágicas, ketamina, special-K, ketolar, imalgene]
- **HEROÍNA** [caballo, jaco]
- **INHALABLES VOLÁTILES** [cola, pegamento, disolvente, poppers, nitritos, gasolina]
- **GHB** [éxtasis líquido, gammahidroxibutirato, X-líquido, gamma-OH]
- **METANFETAMINA** [meth, ice, crystal]
- **SETAS MÁGICAS**

CANNABIS – MARIHUANA – HACHÍS
[aceite de hachís, chocolate, porro, costo, hierba]

Marque con una "X" la casilla (☐) correspondiente a su respuesta
Recuerde que **sólo ha de marcar una casilla** en cada pregunta.

M1. ¿Ha consumido, aunque sólo fuera UNA VEZ EN SU VIDA, CANNABIS, MARIHUANA o HACHÍS (aceite de hachís, chocolate, porro, costo, hierba)**?**

SÍ, HE CONSUMIDO CANNABIS, MARIHUANA o HACHÍS	☐	1
NUNCA HE CONSUMIDO CANNABIS, MARIHUANA o HACHÍS	☐	2

M2. ¿Qué EDAD tenía la PRIMERA VEZ que consumió CANNABIS, MARIHUANA o HACHÍS?
Si no está seguro, anote la edad aproximada. Escriba con números claros sobre la línea o marque el recuadro.

ANOTE LA **EDAD:**__________AÑOS

NUNCA HE CONSUMIDO CANNABIS, MARIHUANA o HACHÍS	☐	1

M3. ¿Cuándo ha sido la PRIMERA VEZ que ha consumido CANNABIS, MARIHUANA o HACHÍS?

HACE UN AÑO O MENOS	☐	1
HACE MÁS DE 1 AÑO	☐	2
NUNCA HE CONSUMIDO CANNABIS, MARIHUANA o HACHÍS	☐	3

M4. ¿Cuántos días ha consumido CANNABIS, MARIHUANA o HACHÍS en los ÚLTIMOS 12 MESES?

Recuerde que 12 meses=52 semanas=365 días

ENTRE 1– 3 DÍAS	☐ 1	ENTRE 30 – 150 DÍAS	☐ 5
ENTRE 4 – 9 DÍAS	☐ 2	MAS DE 150 DÍAS	☐ 6
ENTRE 10 – 19 DÍAS	☐ 3	NO HE CONSUMIDO CANNABIS, MARIHUANA o HACHÍS EN LOS ÚLTIMOS 12 MESES	☐ 7
ENTRE 20 – 29 DÍAS	☐ 4	NUNCA HE CONSUMIDO CANNABIS, MARIHUANA o HACHÍS	☐ 8

M5. ¿Cuántos días ha consumido CANNABIS, MARIHUANA o HACHÍS en los ÚLTIMOS 30 DÍAS?

ENTRE 1– 3 DÍAS	☐ 1	30 DÍAS	☐ 5
ENTRE 4 – 9 DÍAS	☐ 2	NO HE CONSUMIDO CANNABIS, MARIHUANA o HACHÍS EN LOS ÚLTIMOS 30 DÍAS	☐ 6
ENTRE 10 – 19 DÍAS	☐ 3	NUNCA HE CONSUMIDO CANNABIS, MARIHUANA o HACHÍS	☐ 7
ENTRE 20 – 29 DÍAS	☐ 4		

M6. En los ÚLTIMOS 30 DÍAS, cuándo ha consumido CANNABIS, MARIHUANA o HACHÍS, ¿Cuántos PORROS ha fumado EN EL MISMO DÍA por término medio? No considere que fumó porros si lo único que hizo fue dar unas caladas al porro de otra persona.

|___|___| **PORROS**

NO HE CONSUMIDO CANNABIS, MARIHUANA o HACHÍS EN LOS ÚLTIMOS 30 DÍAS	☐ 1
NUNCA HE CONSUMIDO CANNABIS, MARIHUANA o HACHÍS	☐ 2

M7. En los ÚLTIMOS 30 DÍAS, ¿Cuándo ha consumido CANNABIS, MARIHUANA o HACHÍS, qué ha fumado?

PRINCIPALMENTE MARIHUANA (**HIERBA**)	☐ 1
PRINCIPALMENTE HACHÍS (**RESINA, CHOCOLATE**)	☐ 2
DE LOS **2 TIPOS**	☐ 3

NO HE CONSUMIDO CANNABIS, MARIHUANA o HACHÍS EN LOS ÚLTIMOS 30 DÍAS	☐ 4
NUNCA HE CONSUMIDO CANNABIS, MARIHUANA o HACHÍS	☐ 0

M8. En los ÚLTIMOS 30 DÍAS, cuando ha consumido <u>CANNABIS, MARIHUANA o HACHÍS</u>, ¿cómo lo ha consumido? Puede marcar varias opciones.

EN FORMA DE **PORRO** O CANUTO	☐ 1
USANDO PIPAS DE AGUA, BONGS, **CACHIMBAS**, SHISHAS	☐ 2
USANDO **CIGARRILLOS ELECTRÓNICOS**	☐ 3
POR VÍA ORAL A TRAVÉS DE **PASTELES**, GALLETAS, CHUPITOS	☐ 4

NO HE CONSUMIDO CANNABIS, MARIHUANA o HACHÍS EN LOS ÚLTIMOS 30 DÍAS	☐ 9
NUNCA HE CONSUMIDO CANNABIS, MARIHUANA o HACHÍS	☐ 0

M9. En los ÚLTIMOS 30 DÍAS, ¿Cuándo ha consumido <u>CANNABIS, MARIHUANA o HACHÍS</u>, lo ha mezclado con TABACO?

SÍ	☐ 1
NO	☐ 2

NO HE CONSUMIDO CANNABIS, MARIHUANA o HACHÍS EN LOS ÚLTIMOS 30 DÍAS	☐ 1
NUNCA HE CONSUMIDO CANNABIS, MARIHUANA o HACHÍS	☐ 2

M10. Si fuese legal el consumo de <u>CANNABIS, MARIHUANA o HACHÍS</u>, ¿lo probaría?

SÍ	☐ 1
NO	☐ 2

YA LO HE PROBADO	☐ 1

POR FAVOR, RESPONDA A LAS SIGUIENTES PREGUNTAS SI HA CONSUMIDO

CANNABIS, MARIHUANA o HACHÍS EN LOS

ÚLTIMOS 12 MESES

Algunas de las siguientes preguntas le parecerán similares, pero ha de CONTESTARLAS TODAS porque forman parte de cuestionarios internacionales y debemos analizarlas conjuntamente

☐ No he consumido hachís o marihuana en los últimos 12 meses → **Pasa a la pregunta C1**

M11. ¿Con qué frecuencia le ha ocurrido algo de lo que se describe a continuación en los ÚLTIMOS 12 MESES? Ponga una 'X' en un cuadrito de cada fila. En total ha de poner 6 "X", porque hay 6 filas de cuadritos.

	NUNCA	RARAMENTE	DE VEZ EN CUANDO	BASTANTE A MENUDO	MUY A MENUDO
¿Ha fumado cannabis **antes del mediodía**?	☐ 1	☐ 2	☐ 3	☐ 4	☐ 5
¿Ha fumado cannabis **estando solo/a**?	☐ 1	☐ 2	☐ 3	☐ 4	☐ 5
¿Ha tenido **problemas de memoria** al fumar cannabis?	☐ 1	☐ 2	☐ 3	☐ 4	☐ 5
¿Le han dicho los amigos o miembros de tu familia que **deberías reducir el consumo** de cannabis?	☐ 1	☐ 2	☐ 3	☐ 4	☐ 5
¿Ha **intentado reducir** o dejar de consumir cannabis **sin conseguirlo**?	☐ 1	☐ 2	☐ 3	☐ 4	☐ 5
¿Ha tenido **problemas** debido a su consumo de cannabis (disputa, pelea, accidente, mal resultado escolar, bajo rendimiento laboral? ¿Cuáles?: /../	☐ 1	☐ 2	☐ 3	☐ 4	☐ 5

M12. En los ÚLTIMOS 12 MESES ¿ha iniciado algún TRATAMIENTO para dejar o reducir el consumo de CANNABIS, MARIHUANA o HACHÍS (hierba, chocolate, porro, costo, aceite de hachís)?

SÍ	☐ 1
NO	☐ 2

COCAÍNA EN POLVO
[cocaína, perico, farlopa, clorhidrato de cocaína]

Marque con una "X" la casilla (☐) correspondiente a su respuesta
Recuerde que **sólo ha de marcar una casilla** en cada pregunta.

C1. ¿Ha consumido, aunque sólo fuera UNA VEZ EN SU VIDA, COCAÍNA EN POLVO (cocaína, perico, farlopa, clorhidrato de cocaína)**?**

SÍ, HE CONSUMIDO COCAÍNA EN POLVO	☐	1
NUNCA HE CONSUMIDO COCAÍNA EN POLVO	☐	2

C2. ¿Qué EDAD tenía la PRIMERA VEZ que consumió COCAÍNA EN POLVO?
Si no está seguro, anote la edad aproximada. Escriba con números claros sobre la línea o marque el recuadro.

ANOTE LA **EDAD**:___________AÑOS

NUNCA HE CONSUMIDO COCAÍNA EN POLVO	☐	1

C3. ¿Cuándo ha sido la PRIMERA VEZ que ha consumido COCAÍNA EN POLVO?

HACE UN AÑO O MENOS	☐	1
HACE MÁS DE 1 AÑO	☐	2
NUNCA HE CONSUMIDO COCAÍNA EN POLVO	☐	3

C4. ¿Cuántos días ha consumido COCAÍNA EN POLVO en los ÚLTIMOS 12 MESES?

Recuerde que 12 meses=52 semanas=365 días

ENTRE 1– 3 DÍAS	☐	1	ENTRE 30 – 150 DÍAS	☐	5
ENTRE 4 – 9 DÍAS	☐	2	MAS DE 150 DÍAS	☐	6
ENTRE 10 – 19 DÍAS	☐	3	NO HE CONSUMIDO COCAÍNA EN POLVO EN LOS ÚLTIMOS 12 MESES	☐	7
ENTRE 20 – 29 DÍAS	☐	4	NUNCA HE CONSUMIDO COCAÍNA EN POLVO	☐	8

C5. ¿Cuántos días ha consumido COCAÍNA EN POLVO en los ÚLTIMOS 30 DÍAS?

ENTRE 1– 3 DÍAS	☐	1	30 DÍAS	☐	5
ENTRE 4 – 9 DÍAS	☐	2	NO HE CONSUMIDO COCAÍNA EN POLVO EN LOS ÚLTIMOS 30 DÍAS	☐	6
ENTRE 10 – 19 DÍAS	☐	3	NUNCA HE CONSUMIDO COCAÍNA EN POLVO	☐	7
ENTRE 20 – 29 DÍAS	☐	4			

COCAÍNA EN FORMA DE BASE
[base, cocaína cocinada, basuco, boliches, crack, roca]

Marque con una "X" la casilla (☐) correspondiente a su respuesta
Recuerde que **sólo ha de marcar una casilla** en cada pregunta.

B1. Indique si usted ha consumido <u>COCAÍNA EN FORMA DE BASE</u> ALGUNA VEZ EN LA VIDA, y/o en los ÚLTIMOS 12 MESES y/o en los ÚLTIMOS 30 DÍAS. Si ha consumido anote en el recuadro correspondiente la EDAD en que la consumió por PRIMERA VEZ.

	¿Ha consumido ALGUNA VEZ EN LA VIDA?		¿Ha consumido alguna vez en los ÚLTIMOS 12 MESES?		¿Ha consumido alguna vez en los ÚLTIMOS 30 DÍAS?		EDAD en que consumió <u>por primera vez en su vida</u>
	No	Sí	No	Sí	No	Sí	
**COCAÍNA EN FORMA DE BASE** [base, cocaína cocinada, basuco, boliches, crack, roca]	☐ 1	☐ 2	☐ 1	☐ 2	☐ 1	☐ 2	

B2. ¿Cuándo ha sido la PRIMERA VEZ que ha consumido COCAÍNA EN FORMA DE BASE?

HACE UN AÑO O MENOS	☐	1
HACE MÁS DE 1 AÑO	☐	2
NUNCA HE CONSUMIDO COCAÍNA EN FORMA DE BASE	☐	3

ÉXTASIS U OTRAS DROGAS DE SÍNTESIS
[pastis, pirulas, cristal, MDMA]

Marque con una "X" la casilla (☐) correspondiente a su respuesta
Recuerde que **sólo ha de marcar una casilla** en cada pregunta.

E1. Indique si usted ha consumido <u>ÉXTASIS</u> ALGUNA VEZ EN LA VIDA, y/o en los ÚLTIMOS 12 MESES y/o en los ÚLTIMOS 30 DÍAS. Si ha consumido anote en el recuadro correspondiente la EDAD en que la consumió por PRIMERA VEZ.

	¿Ha consumido ALGUNA VEZ EN LA VIDA?		¿Ha consumido alguna vez en los ÚLTIMOS 12 MESES?		¿Ha consumido alguna vez en los ÚLTIMOS 30 DÍAS?		EDAD en que consumió <u>por primera vez en su vida</u>
	No	Sí	No	Sí	No	Sí	
**ÉXTASIS U OTRAS DROGAS DE SÍNTESIS** (pastis, pirulas, crista, MDMAl)	☐ 1	☐ 2	☐ 1	☐ 2	☐ 1	☐ 2	

ANFETAMINAS O SPEED
[anfetas]

Marque con una "X" la casilla (☐) correspondiente a su respuesta
Recuerde que **sólo ha de marcar una casilla** en cada pregunta.

Sp1. Indique si usted ha consumido <u>ANFETAMINAS O SPEED</u> ALGUNA VEZ EN LA VIDA, y/o en los ÚLTIMOS 12 MESES y/o en los ÚLTIMOS 30 DÍAS. Si ha consumido anote en el recuadro correspondiente la EDAD en que la consumió por PRIMERA VEZ.

	¿Ha consumido ALGUNA VEZ EN LA VIDA?		¿Ha consumido alguna vez en los ÚLTIMOS 12 MESES?		¿Ha consumido alguna vez en los ÚLTIMOS 30 DÍAS?		EDAD en que consumió <u>por primera vez en su vida</u>
	No	Sí	No	Sí	No	Sí	
**ANFETAMINAS O SPEED** (anfetas)	☐1	☐2	☐1	☐2	☐1	☐2	

ALUCINÓGENOS
[LSD, ácido, tripi, setas mágicas, ketamina, special-K, ketolar, imalgene]

Marque con una "X" la casilla (☐) correspondiente a su respuesta
Recuerde que **sólo ha de marcar una casilla** en cada pregunta.

Alu1. Indique si usted ha consumido <u>ALUCINÓGENOS</u> ALGUNA VEZ EN LA VIDA, y/o en los ÚLTIMOS 12 MESES y/o en los ÚLTIMOS 30 DÍAS. Si ha consumido anote en el recuadro correspondiente la EDAD en que la consumió por PRIMERA VEZ.

	¿Ha consumido ALGUNA VEZ EN LA VIDA?		¿Ha consumido alguna vez en los ÚLTIMOS 12 MESES?		¿Ha consumido alguna vez en los ÚLTIMOS 30 DÍAS?		EDAD en que consumió <u>por primera vez en su vida</u>
	No	Sí	No	Sí	No	Sí	
**ALUCINÓGENOS** (LSD, ácido, tripi, setas mágicas, ketamina, special-K, ketolar, imalgene)	☐1	☐2	☐1	☐2	☐1	☐2	

HEROÍNA
[caballo, jaco]

Marque con una "X" la casilla (☐) correspondiente a su respuesta
Recuerde que **sólo ha de marcar una casilla** en cada pregunta.

H1. Indique si usted ha consumido <u>HEROÍNA</u> ALGUNA VEZ EN LA VIDA, y/o en los ÚLTIMOS 12 MESES y/o en los ÚLTIMOS 30 DÍAS. Si ha consumido anote en el recuadro correspondiente la EDAD en que la consumió por PRIMERA VEZ.

	¿Ha consumido ALGUNA VEZ EN LA VIDA?		¿Ha consumido alguna vez en los ÚLTIMOS 12 MESES?		¿Ha consumido alguna vez en los ÚLTIMOS 30 DÍAS?		EDAD en que consumió <u>por primera vez en su vida</u>
	No	Sí	No	Sí	No	Sí	
**HEROÍNA** (caballo, jaco)	☐1	☐2	☐1	☐2	☐1	☐2	

INHALABLES VOLÁTILES
[cola, pegamento, disolvente, poppers, nitritos, gasolina]

Marque con una "X" la casilla (☐) correspondiente a su respuesta
Recuerde que **sólo ha de marcar una casilla** en cada pregunta.

InV1. Indique si usted ha consumido <u>INHALABLES VOLÁTILES</u> ALGUNA VEZ EN LA VIDA, y/o en los ÚLTIMOS 12 MESES y/o en los ÚLTIMOS 30 DÍAS. Si ha consumido anote en el recuadro correspondiente la EDAD en que la consumió por PRIMERA VEZ.

	¿Ha consumido ALGUNA VEZ EN LA VIDA?		¿Ha consumido alguna vez en los ÚLTIMOS 12 MESES?		¿Ha consumido alguna vez en los ÚLTIMOS 30 DÍAS?		EDAD en que consumió <u>por primera vez en su vida</u>
	No	Sí	No	Sí	No	Sí	
**INHALABLES VOLÁTILES** (cola, pegamento, disolvente, poppers, nitritos, gasolina)	☐1	☐2	☐1	☐2	☐1	☐2	

GHB
[éxtasis líquido, gammahidroxibutirato, X-líquido, gamma-OH]

Marque con una "X" la casilla (☐) correspondiente a su respuesta
Recuerde que **sólo ha de marcar una casilla** en cada pregunta.

GHB1. Indique si usted ha consumido <u>GHB</u> ALGUNA VEZ EN LA VIDA, y/o en los ÚLTIMOS 12 MESES y/o en los ÚLTIMOS 30 DÍAS. Si ha consumido anote en el recuadro correspondiente la EDAD en que la consumió por PRIMERA VEZ.

	¿Ha consumido ALGUNA VEZ EN LA VIDA?		¿Ha consumido alguna vez en los ÚLTIMOS 12 MESES?		¿Ha consumido alguna vez en los ÚLTIMOS 30 DÍAS?		EDAD en que consumió <u>por primera vez en su vida</u>
	No	Sí	No	Sí	No	Sí	
**GHB** (éxtasis líquido, gammahidroxibutirato, X-líquido, gamma-OH)	☐1	☐2	☐1	☐2	☐1	☐2	

METANFETAMINA
[meth, ice, crystal]

Marque con una "X" la casilla (☐) correspondiente a su respuesta
Recuerde que **sólo ha de marcar una casilla** en cada pregunta.

MET1. Indique si usted ha consumido <u>METANFETAMINA</u> ALGUNA VEZ EN LA VIDA, y/o en los ÚLTIMOS 12 MESES y/o en los ÚLTIMOS 30 DÍAS. Si ha consumido anote en el recuadro correspondiente la EDAD en que la consumió por PRIMERA VEZ.

	¿Ha consumido ALGUNA VEZ EN LA VIDA?		¿Ha consumido alguna vez en los ÚLTIMOS 12 MESES?		¿Ha consumido alguna vez en los ÚLTIMOS 30 DÍAS?		EDAD en que consumió por primera vez en su vida
	No	Sí	No	Sí	No	Sí	
**METANFETAMINA** (meth, ice, crystal)	☐1	☐2	☐1	☐2	☐1	☐2	

SETAS MÁGICAS

Marque con una "X" la casilla (☐) correspondiente a su respuesta
Recuerde que **sólo ha de marcar una casilla** en cada pregunta.

SM1. Indique si usted ha consumido <u>SETAS MÁGICAS</u> ALGUNA VEZ EN LA VIDA, y/o en los ÚLTIMOS 12 MESES y/o en los ÚLTIMOS 30 DÍAS. Si ha consumido anote en el recuadro correspondiente la EDAD en que la consumió por PRIMERA VEZ.

	¿Ha consumido ALGUNA VEZ EN LA VIDA?		¿Ha consumido alguna vez en los ÚLTIMOS 12 MESES?		¿Ha consumido alguna vez en los ÚLTIMOS 30 DÍAS?		EDAD en que consumió por primera vez en su vida
	No	Sí	No	Sí	No	Sí	
**SETAS MÁGICAS**	☐1	☐2	☐1	☐2	☐1	☐2	

DROGAS INYECTADAS

Marque con una "X" la casilla (☐) correspondiente a su respuesta
Recuerde que sólo ha de marcar una casilla en cada pregunta.

Iny1. ¿Se ha inyectado Vd. ALGUNA VEZ EN LA VIDA HEROÍNA, COCAÍNA U OTRAS DROGAS ILEGALES?

SÍ	☐	1
NO, NUNCA ME HE INYECTADO HEROÍNA, COCAÍNA O DROGAS ILEGALES	☐	2

Iny2. En los ÚLTIMOS 12 MESES, ¿Se ha inyectado Vd. alguna vez HEROÍNA, COCAÍNA U OTRAS DROGAS ILEGALES?

SÍ	☐	1
NO ME HE INYECTADO HEROÍNA, COCAÍNA U OTRAS DROGAS ILEGALES EN LOS ÚLTIMOS 12 MESES	☐	2
NUNCA ME HE INYECTADO HEROÍNA, COCAÍNA U OTRAS DROGAS ILEGALES	☐	3

Iny3. Si se ha inyectado en los ÚLTIMOS 12 MESES, HEROÍNA, COCAÍNA U OTRAS DROGAS ILEGALES ¿Ha compartido alguna vez con otra persona jeringuillas o cualquier otro material de inyección (toallitas, cucharas, gomas…)?

SÍ HE COMPARTIDO JERINGUILLAS U OTRO MATERIAL DE INYECCIÓN	☐	1
NO HE COMPARTIDO JERINGUILLAS U OTRO MATERIAL DE INYECCIÓN	☐	2
NUNCA ME HE INYECTADO HEROÍNA, COCAÍNA U OTRAS DROGAS ILEGALES EN LOS ÚLTIMOS 12 MESES	☐	3

NUEVAS SUSTANCIAS

Marque con una "X" la casilla (☐) correspondiente a su respuesta
Recuerde que **sólo ha de marcar una casilla** en cada pregunta.

NS1. En la actualidad se encuentran disponibles NUEVAS sustancias que imitan el efecto de drogas ilegales (cannabis, cocaína, éxtasis….). Estas nuevas sustancias (keta, spice, cannabinoides sintéticos, marihuana sintética, miau miau, flakka, superman, catinonas, mefedrona, derivados de los fentanilos, metoxetamina, NBOMe, ayahuasca, kratom…) se pueden presentar en forma de hierbas, pastillas, polvos, líquidos, inciensos, etc… Conteste las siguientes preguntas referentes a estas nuevas sustancias.

¿Ha oído hablar de estas nuevas sustancias?		¿Las ha consumido ALGUNA VEZ EN LA VIDA?		¿Las ha consumido alguna vez en los ÚLTIMOS 12 MESES?		¿Las ha consumido alguna vez en los ÚLTIMOS 30 DÍAS?	
No	Sí	No	Sí	No	Sí	No	Sí
☐1	☐2	☐1	☐2	☐1	☐2	☐1	☐2

NS2. ¿Dónde consiguió o le ofrecieron este tipo de sustancias, EN LOS ÚLTIMOS 12 MESES? Puede marcar una o varias opciones.

ME LA OFRECIÓ UN **AMIGO**	☐	1
LAS COMPRÉ EN UNA **TIENDA ESPECIALIZADA** (SMART SHOP O HEAD SHOP)	☐	2
A TRAVÉS DE UN **VENDEDOR** (Camello)	☐	3
A TRAVÉS DE **INTERNET**	☐	4
ME LA OFRECIERON EN UNA **FIESTA**, EN UN PUB, EN UN BAR	☐	5
OTROS SITIOS	☐	6
NO HE CONSUMIDO O NO ME HA OFRECIDO ESTE TIPO DE SUSTANCIAS	☐	7

NS3. Para cada una de las siguientes sustancias, indique si usted las has consumido ALGUNA VEZ EN LA VIDA, y/o en los ÚLTIMOS 12 MESES y/o en los ÚLTIMOS 30 DÍAS. Si ha consumido, anote en el recuadro correspondiente la EDAD en que la consumió por PRIMERA VEZ.

	¿Ha consumido ALGUNA VEZ EN LA VIDA?		¿Ha consumido alguna vez en los ÚLTIMOS 12 MESES?		¿Ha consumido alguna vez en los ÚLTIMOS 30 DÍAS?		EDAD en que consumió por primera vez en su vida	¿La ha conseguido por internet?	
	No	Sí	No	Sí	No	Sí		No	SI
**KETAMINA** (también llamada el K, Ketolar, Special K, vitamina K, polvo k)	☐1	☐2	☐1	☐2	☐1	☐2		☐1	☐2
**SPICE** (también llamada spice drugs, cannabinoides sintéticos, marihuana sintética)	☐1	☐2	☐1	☐2	☐1	☐2		☐1	☐2
**MEFEDRONA** (también llamada miau-miau, 4-MMC, burbujas azules, catmef)	☐1	☐2	☐1	☐2	☐1	☐2		☐1	☐2
**SALVIA DIVINORUM** (también llamada ska pastora, ska María, hierba María o hierba de los Dioses, Sally D, Lady Salvia, Magic Mint)	☐1	☐2	☐1	☐2	☐1	☐2		☐1	☐2
**AYAHUASCA**	☐1	☐2	☐1	☐2	☐1	☐2		☐1	☐2

VD. HA TERMINADO LAS PREGUNTAS ACERCA DE NUEVAS SUSTANCIAS. GRACIAS.
POR FAVOR, AHORA CONTINUE LEYENDO Y RESPONDIENDO POR VD. MISMO LAS PREGUNTAS QUE SIGUEN Y SI TIENE ALGUNA DUDA PUEDE CONSULTAR AL ENTREVISTADOR/A EN CUALQUIER MOMENTO

TRATAMIENTO

Marque con una "X" la casilla (☐) correspondiente a su respuesta
Recuerde que **sólo ha de marcar una casilla** en cada pregunta.

TR1. ¿Conoce personalmente a alguien que haya consumido HEROÍNA O SE HAYA INYECTADO DROGAS ILEGALES en los ÚLTIMOS 12 MESES?

SÍ, CONOZCO A ALGUIEN	☐	1
NO, NO CONOZCO A NADIE	☐	2

TR2. Por favor, invente un nombre para cada persona que conozca personalmente y que sabe que ha consumido HEROÍNA O SE HA INYECTADO DROGAS ILEGALES en los ÚLTIMOS 12 MESES, y luego para cada una de ellas, conteste las preguntas de la siguiente tabla.
Si tiene dificultades para rellenarla, pídale ayuda al entrevistador. Él podrá explicarle mejor las instrucciones.

Nombre inventado		¿Se ha inyectado drogas ilegales en los ÚLTIMOS 12 MESES?			¿Ha consumido heroína en los ÚLTIMOS 12 MESES?			¿Ha iniciado un tratamiento ambulatorio por drogas ilegales en los ÚLTIMOS 12 MESES?			¿Estuvo motivado dicho tratamiento por la heroína?			¿Ha estado en mantenimiento con metadona en los ÚLTIMOS 12 MESES?		
		SÍ	NO	NO SABE	SÍ	NO	NO SABE	SÍ	NO	NO SABE	SÍ	NO	NO SABE	SÍ	NO	NO SABE
01	____________	☐1	☐2	☐3	☐1	☐2	☐3	☐1	☐2	☐3	☐1	☐2	☐3	☐1	☐2	☐3
02	____________	☐1	☐2	☐3	☐1	☐2	☐3	☐1	☐2	☐3	☐1	☐2	☐3	☐1	☐2	☐3
03	____________	☐1	☐2	☐3	☐1	☐2	☐3	☐1	☐2	☐3	☐1	☐2	☐3	☐1	☐2	☐3
04	____________	☐1	☐2	☐3	☐1	☐2	☐3	☐1	☐2	☐3	☐1	☐2	☐3	☐1	☐2	☐3
05	____________	☐1	☐2	☐3	☐1	☐2	☐3	☐1	☐2	☐3	☐1	☐2	☐3	☐1	☐2	☐3

No conozco personalmente a nadie que haya consumido heroína o se haya inyectado drogas ilegales en los ÚLTIMOS 12 MESES	☐1

JUEGO

J1. En LOS ÚLTIMOS 12 MESES ¿Con qué frecuencia HA JUGADO DINERO? Marque una casilla por columna.

	EN INTERNET	FUERA DE INTERNET
UN DÍA AL MES o MENOS	☐ 1	☐ 1
2-4 DÍAS AL MES	☐ 2	☐ 2
2-3 DÍAS A LA SEMANA	☐ 3	☐ 3
4-5 DÍAS A LA SEMANA	☐ 4	☐ 4
6 o MÁS DÍAS A LA SEMANA	☐ 5	☐ 5

NO HE JUGADO DINERO EN LOS ÚLTIMOS 12 MESES	☐ 0	☐ 0
NUNCA HE JUGADO DINERO	☐ 9	☐ 9

J2. ¿Qué edad tenía la PRIMERA VEZ que jugó dinero?
Ponga sobre las líneas los años que tenía o marque el recuadro correspondiente.

ANOTE LA **EDAD** que tenía la **primera vez** que **JUGÓ DINERO EN INTERNET:**_______AÑOS

NUNCA HE JUGADO DINERO EN INTERNET ☐ 1

ANOTE LA **EDAD** que tenía la **primera vez** que **JUGÓ DINERO FUERA DE INTERNET**:_______AÑOS

NUNCA HE JUGADO DINERO FUERA DE INTERNET ☐ 1

J3. En LOS ÚLTIMOS 12 MESES ¿En qué juegos HA JUGADO DINERO? Marque una o varias casillas.

	EN INTERNET	FUERA DE INTERNET
LOTERÍAS, PRIMITIVA, BONOLOTO, ONCE CUPONES, JUEGO ACTIVOEUROJACKPOT, 7/39	☐ 1	☐ 1
LOTERÍAS INSTANTÁNEAS (RASCAS ONCE)	☐ 2	☐ 2
QUINIELAS DE FÚTBOL Y/O QUINIGOL	☐ 3	☐ 3
APUESTAS DEPORTIVAS	☐ 4	☐ 4
APUESTAS EN LAS CARRERAS DE CABALLOS	☐ 5	☐ 5
SLOTS, MÁQUINAS DE AZAR/ TRAGAPERRAS	☐ 6	☐ 6
JUEGOS DE CARTAS CON DINERO (PÓQUER, MUS, BLACKJACK, PUNTO Y BANCA…)	☐ 7	☐ 7
BINGO	☐ 8	☐ 8
VIDEOJUEGOS	☐ 9	☐ 9
JUEGOS EN CASINO	☐ 10	☐ 10
JUEGOS EN SALAS DE JUEGO	☐ 11	☐ 11
BOLSA DE VALORES	☐ 12	☐ 12
CONCURSOS (TELEVISIÓN, RADIO, PRENSA) CON APUESTA ECONÓMICA	☐ 13	☐ 13

NO HE JUGADO DINERO EN LOS ÚLTIMOS 12 MESES	☐ 19	☐ 19
NUNCA HE JUGADO DINERO	☐ 20	☐ 20

J4. En los ÚLTIMOS 12 MESES, ¿Cuál ha sido la mayor cantidad de DINERO QUE SE HA GASTADO JUGANDO en un solo día? Marque una casilla por columna.

	**Dinero que he jugado EN INTERNET**	**Dinero que he jugado FUERA DE INTERNET**
MENOS DE 6 EUROS	☐1	☐1
ENTRE 6 Y 30 EUROS	☐2	☐2
ENTRE 31 Y 60 EUROS	☐3	☐3
ENTRE 61 Y 300 EUROS	☐4	☐4
MÁS DE 300 EUROS	☐5	☐5

NO HE JUGADO DINERO EN LOS ÚLTIMOS 12 MESES	☐0	☐0
NUNCA HE JUGADO DINERO	☐9	☐9

Ahora vamos a volver a preguntarle algunas cosas sobre **JUGAR CON DINERO EN LOS 12 ÚLTIMOS MESES.**

Muy importante:

- SI NO HA JUGADO CON DINERO EN LOS ÚLTIMOS 12 MESES, no conteste este bloque y pase a la pregunta INT1 (Internet)

- SI HA JUGADO CON DINERO EN LOS ÚLTIMOS 12 MESES ya sea en internet o fuera de internet, DEBE CONTESTAR TODAS LAS PREGUNTAS porque forman parte de un cuestionario y deben analizarse conjuntamente.

J5. ¿Conteste si le ha ocurrido (sí/no) algo de lo que se describe a continuación en los ÚLTIMOS 12 MESES? Marque una casilla por línea.

	**SÍ**	**NO**
HA TENIDO LA NECESIDAD DE APOSTAR **CANTIDADES DE DINERO CADA VEZ MAYORES** PARA CONSEGUIR LA EXCITACIÓN DESEADA.	☐1	☐2
HA ESTADO **NERVIOSO O IRRITADO** CUANDO HA **INTENTADO REDUCIR** O ABANDONAR EL JUEGO.	☐1	☐2
HA HECHO ESFUERZOS REPETIDOS PARA **CONTROLAR, REDUCIR O ABANDONAR** EL JUEGO, SIEMPRE SIN ÉXITO.	☐1	☐2
A MENUDO HA TENIDO LA **MENTE OCUPADA EN LAS APUESTAS** (P. EJ. REVIVIENDO EXPERIENCIAS DE APUESTAS PASADAS, PLANIFICANDO SU PRÓXIMA APUESTA, PENSANDO EN FORMAS DE CONSEGUIR DINERO PARA APOSTAR…).	☐1	☐2
A MENUDO HA **JUGADO CUANDO SENTÍA DESASOSIEGO** (P. EJ. CULPABILIDAD, ANSIEDAD, DEPRESIÓN).	☐1	☐2
DESPUÉS DE PERDER DINERO EN LAS APUESTAS, HA VUELTO A **JUGAR OTRO DÍA PARA INTENTAR GANAR** Y ASÍ RECUPERAR EL DINERO PERDIDO.	☐1	☐2
HA **MENTIDO** PARA OCULTAR SU GRADO DE IMPLICACIÓN EN EL JUEGO.	☐1	☐2
**HA PUESTO EN PELIGRO O HA PERDIDO** ALGUNA **RELACIÓN** IMPORTANTE, SU **EMPLEO** U OPORTUNIDADES EN SUS ESTUDIOS O EN SU CARRERA PROFESIONAL A CAUSA DEL JUEGO.	☐1	☐2
CUENTA CON LOS DEMÁS PARA QUE **LE DEN DINERO** PARA ALIVIAR SU GRAVE SITUACIÓN FINANCIERA PROVOCADA POR EL JUEGO,	☐1	☐2

INTERNET

INT1. Por favor, lea las afirmaciones siguientes respecto al uso de INTERNET (chateando, mandando o recibiendo e-mails, whatsApps, usando redes sociales, jugando con o sin dinero, escuchando o descargándote música, viendo o descargándose videos…). Indique cada cuanto tiempo le ocurren las siguientes situaciones. (No tenga en cuenta el tiempo que emplea en internet para hacer deberes o trabajar, sólo indique aquí el tiempo que emplea en internet por diversión). Marque una casilla por línea.

	NUNCA	RARA VEZ	ALGUNA VEZ	A MENUDO	MUY FRECUENTEMENTE
¿Con qué frecuencia le ha resultado difícil **dejar de usar internet** cuando estaba conectado?	☐0	☐1	☐2	☐3	☐4
¿Con qué frecuencia ha seguido **conectado** a internet **a pesar de querer parar**?	☐0	☐1	☐2	☐3	☐4
¿Con qué frecuencia sus padres, o amigos le dicen que **debería pasar menos tiempo** en internet?	☐0	☐1	☐2	☐3	☐4
¿Con qué frecuencia **prefiere conectarse a internet** en vez de pasar el tiempo con otros (padres, amigos…)?	☐0	☐1	☐2	☐3	☐4
¿Con qué frecuencia **duerme menos** por estar conectado a internet?	☐0	☐1	☐2	☐3	☐4
¿Con qué frecuencia se encuentra **pensando en internet**, aunque no esté conectado?	☐0	☐1	☐2	☐3	☐4
¿Con qué frecuencia **está deseando conectarse** a internet?	☐0	☐1	☐2	☐3	☐4
¿Con qué frecuencia piensa que **debería usar menos internet**?	☐0	☐1	☐2	☐3	☐4
¿Con qué frecuencia **ha intentado pasar menos tiempo** conectado a internet y no lo ha conseguido?	☐0	☐1	☐2	☐3	☐4
¿Con qué frecuencia intenta **terminar su trabajo a toda prisa** para conectarse a internet?	☐0	☐1	☐2	☐3	☐4
¿Con qué frecuencia **descuida sus obligaciones** (deberes, estar con la familia…) porque prefiere conectarse a internet?	☐0	☐1	☐2	☐3	☐4
¿Con qué frecuencia **se conecta** a internet cuando **estas "de bajón"**?	☐0	☐1	☐2	☐3	☐4
¿Con qué frecuencia **se conecta** a internet para **olvidar sus penas o sentimientos negativos**?	☐0	☐1	☐2	☐3	☐4
¿Con qué frecuencia se siente **inquieto, frustrado o irritado si no puede usar internet**?	☐0	☐1	☐2	☐3	☐4
¿Con qué frecuencia se ha sentido **acosado, amenazado** o cree que le han hecho **bullying** a través de internet?	☐0	☐1	☐2	☐3	☐4

SALUD

S1. Señale si ALGUNA VEZ EN LA VIDA el consumo de medicamentos, alcohol u otras drogas le han producido a Vd. alguno de los siguientes PROBLEMAS.
Marque todas las que considere.

PELEAS O PROBLEMAS CON **FAMILIARES, AMIGOS,** COMPAÑEROS O VECINOS	☐	1
PROBLEMAS DE **SALUD FÍSICA** (ARRITMIAS, DESMAYO, ASMA) O NECESIDAD DE ACUDIR A UN CENTRO DE URGENCIAS	☐	2
PROBLEMAS **PSICOLÓGICOS** O PSIQUIÁTRICOS (DEPRESIÓN, ANSIEDAD, ESQUIZOFRENIA, PARANOIA, CRISIS DE PÁNICO...)	☐	3
**ACCIDENTES DE TRÁFICO**	☐	4
PROBLEMAS **LABORALES** (ACCIDENTES, DESPIDOS O SANCIONES…)	☐	5
PROBLEMAS **ECONÓMICOS**	☐	6
PROBLEMAS CON LA **POLICÍA O LA LEY**	☐	7
RELACIONES **SEXUALES** QUE EN OTRAS CIRCUNSTANCIAS NO HUBIERA MANTENIDO	☐	8
RELACIONES **SEXUALES SIN PROTECCIÓN**	☐	9
HE SIDO VÍCTIMA DE UNA SITUACIÓN DE **VIOLENCIA DE GÉNERO** POR MI PAREJA O EXPAREJA	☐	10
NUNCA HE TENIDO PROBLEMAS DE ESTE TIPO	☐	11
NUNCA HE CONSUMIDO ALCOHOL U OTRAS DROGAS	☐	12

S2. EN ALGÚN MOMENTO DE SU VIDA, ha sido Vd. diagnosticado de:
Marque todas las que considere.

LUDOPATÍA (PROBLEMAS CON EL JUEGO)	☐	1
ANSIEDAD	☐	2
DEPRESIÓN	☐	3
INSOMNIO (PROBLEMAS PARA DORMIR)	☐	4
TDAH (TRASTORNO POR DEFICIT DE ATENCIÓN CON HIPERACTIVIDAD)	☐	5
ESQUIZOFRENIA U OTRAS ENFERMEDADES SIMILARES	☐	6
TRASTORNO BIPOLAR	☐	7
TRASTORNO DE PERSONALIDAD	☐	8
TRASTORNOS DE LA ALIMENTACIÓN (ANOREXIA, BULIMIA)	☐	9
VIRUS DEL SIDA (VIH)	☐	10
VIRUS DE LA HEPATITIS B	☐	11
VIRUS DE LA HEPATITIS C	☐	12
NINGUNO DE LOS MENCIONADOS	☐	13
NO DESEO CONTESTAR	☐	14

USTED HA TERMINADO EL CUESTIONARIO AUTORRELLENABLE.
INTRODÚZCALO EN EL SOBRE Y ENTRÉGUESELO AL ENTREVISTADOR/A.
GRACIAS POR SU COLABORACIÓN.

ENTREVISTADOR/A: PASAR A BLOQUE DE PREGUNTAS DE “RIESGO Y OTRAS CUESTIONES” EN EL CUADERNILLO DEL ENTREVISTADOR/A
