# Supplementary material for: Excessive Drinking Among Men Who Have Sex With Men Recruited From Web-Based Resources: Cross-sectional Questionnaire Study
Source: JMIR Public Health Surveill. 2022 Oct 31;8(10):e32888. doi: 10.2196/32888 (PMC9664322; doi:10.2196/32888)
Supplement: Multimedia Appendix 3 [file publichealth_v8i10e32888_app3.pdf]

*** 1. To participate, please confirm the following requisite:**

- [ ] On my age, I am legally authorized to have sexual relations and I want to participate in the study.

Before to start REMEMBER:

- To advance in the questionnaire use the buttons PREVIOUS and NEXT that are placed after each question.
- DO NOT use the "back" button in your navigator, doing so you may abandon the survey. If so, refresh the site and you will obtain a message. Press continue to go return to the survey.

*** 1. Para participar por favor, marca la siguiente casilla:**

- [ ] Tengo la edad legal para tener relaciones sexuales y quiero participar en el estudio.

Antes de empezar **RECUERDA**:

- Para moverte por la encuesta usa siempre los botones de ANTERIOR y SIGUIENTE que tienes después de cada pregunta.
- **NO** uses el botón de retroceso del móvil, te puede sacar de la encuesta. Si te pasa, refresca el navegado y te saldrá un aviso *"Confirmar reenvío del formulario"*. Dale a **continuar** para regresar a la encuesta.
