# Supplementary material for: Excessive Drinking Among Men Who Have Sex With Men Recruited From Web-Based Resources: Cross-sectional Questionnaire Study
Source: JMIR Public Health Surveill. 2022 Oct 31;8(10):e32888. doi: 10.2196/32888 (PMC9664322; doi:10.2196/32888)
Supplement: Multimedia Appendix 4 [file publichealth_v8i10e32888_app4.pdf]

Table S1. Comparison of prevalence of three AUDIT measures between men who have sex with men (MSM) and general population men (GPM), by age, education level, size of place of residence and other drug use. Spain, 2018-2020.

	aPR [95% CI] (Model I)[a]	aPR [95% CI] (Model II)[b]
**Hazardous/harmful drinking (AUDIT score ≥8)**		
**All participants**	1.8 [1.6-2.0]	1.2 [1.0-1.4]*
**Age**		
16-24	1.5 [1.2-2.0]	1.2 [1.0-1.6]
25-34	1.8 [1.5-2.3]	1.2 [1.0-1.5]
35-64	1.8 [1.5-2.2]	1.1 [0.9-1.4]
**Education level**		
No university	1.8 [1.5-2.1]	1.2 [1.0-1.4]*
University	1.7 [1.3-2.3]	1.1 [0.8-1.5]
**Size of place of residence (inhabitants)**		
<10,000	1.5 [1.1-2.0]	1.0 [0.7-1.4]
10,000-500,000	1.8 [1.5-2.1]	1.1 [0.9-1.4]
>500,000	2.2 [1.7-2.9]	1.5 [1.1-1.9]
**Other drug use in past 12 months**		
No	0.9 [0.7-1.3]	0.9 [0.7-1.3]
Yes	1.2 [1.0-1.5]*	1.2 [1.0-1.5]*
**Hazardous drinking (AUDIT-C score ≥4)**		
**All participants**	0.9 [0.9-1.0]	0.8 [0.7-0.8]
**Age**		
16-24	1.1 [0.9-1.3]	0.9 [0.8-1.1]
25-34	1.1 [1.0-1.3]	0.9 [0.8-1.0]*
35-64	0.9 [0.8-0.9]	0.7 [0.6-0.8]
**Education level**		
No university	0.9 [0.8-1.0]*	0.8 [0.7-0.8]
University	1.0 [0.9-1.1]	0.8 [0.7-0.8]
**Size of place of residence (inhabitants)**		
<10,000	0.8 [0.7-0.9]	0.7 [0.6-0.8]
10,000-500,000	1.0 [0.9-1.0]	0.8 [0.7-0.8]
>500,000	1.0 [0.9-1.2]	0.8 [0.7-0.9]
**Other drug use in past 12 months**		
No	0.6 [0.5-0.7]	0.6 [0.5-0.7]
Yes	0.9 [0-8-0.9]	0.9 [0.8-0.9]
**Harmful drinking (AUDIT-P score ≥4)**		
**All participants**	2.3 [2.0-2.7]	1.5 [1.3-1.7]
**Age**		
16-24	2.3 [1.7-3.1]	1.8 [1.3-2.4]
25-34	2.1 [1.6-2.7]	1.3 [1.0-1.7]
35-64	2.4 [1.9-3.0]	1.4 [1.1-1.8]
**Education level**		
No university	2.2 [1.8-2.6]	1.4 [1.2-1.7]
University	2.6 [1.7-3.8]	1.6 [1.1-2.5]
**Size of place of residence (inhabitants)**		
<10,000	1.8 [1.2-2.6]	1.1 [0.8-1.7]
10,000-500,000	2.1 [1.7-2.6]	1.4 [1.1-1.7]
>500,000	3.4 [2.4-4.7]	2.2 [1.6-3.0]
**Other drug use in past 12 months**		
No	1.4 [1.0-2.0]*	1.4 [1.0-2.0]*
Yes	1.5 [1.2-1.8]	1.5 [1.2-1.8]

[a]aPR were obtained from Poisson regression with robust variance in the framework of generalized linear models and adjusted by age, education level, size of place of residence, country of birth and economic status. The reference group (aPR=1) was general population men.

[b]including adjustment for covariates in Model I plus other drug use in past 12 months.

**P* <.05

Table S2. Comparison of prevalence of regular hazardous drinking and monthly binge drinking between men who have sex with men (MSM) and general population men (GPM), by age, education level, size of place of residence and other drug use. Spain, 2018-2020.

	**aPR [95% CI] (Model I)[a]**	**aPR [95% CI] (Model II)[b]**
**Regular hazardous drinking (>14 drinks/week)**		
**All participants**	0.7 [0.6-0.9]	0.5 [0.4-0.6]
**Age**		
16-24	0.6 [0.3-1.2]	0.5 [0.2-0.9]
25-34	0.9 [0.6-1.4]	0.6 [0.4-0.9]
35-64	0.6 [0.5-0.8]	0.5 [0.3-0.6]
**Education level**		
No university	0.6 [0.5-0.8]	0.4 [0.3-0.6]
University	0.8 [0.5-1.2]	0.5 [0.3-0.8]
**Size of place of residence (inhabitants)**		
<10,000	0.3 [0.2-0.6]	0.3 [0.1-0.5]
10,000-500,000	0.6 [0.5-0.9]	0.4 [0.3-0.6]
>500,000	1.1 [0.7-1.7]	0.8 [0.5-1.2]
**Other drug use in past 12 months**		
No	0.3 [0.2-0.6]	0.3 [0.2-0.6]
Yes	0.5 [0.4-0.6]	0.5 [0.4-0.6]
**Binge drinking (at least monthly)**		
**All participants**	1.7 [1.5-1.9]	1.2 [1.0-1.3]
**Age**		
16-24	1.7 [1.2-2.3]	1.4 [1.0-1.8]
25-34	1.7 [1.3-2.1]	1.3 [1.0-1.6]
35-64	1.6 [1.3-2.0]	1.0 [0.8-1.3]
**Education level**		
No university	1.6 [1.4-1.9]	1.2 [1.0-1.4]
University	1.8 [1.3-2.4]	1.1 [0.8-1.6]
**Size of place of residence (inhabitants)**		
<10,000	1.4 [1.0-1.9]*	1.1 [0.8-1.5]
10,000-500,000	1.7 [1.4-2.1]	1.1 [0.9-1.4]
>500,000	1.8 [1.4-2.4]	1.3 [1.0-1.8]
**Other drug use in past 12 months**		
No	1.0 [0.7-1.3]	1.0 [0.7-1.3]
Yes	1.2 [1.0-1.4]	1.2 [1.0-1.4]

[a]aPR were obtained from Poisson regression with robust variance in the framework of generalized linear models and adjusted by age, education level, size of place of residence, country of birth and economic status. The reference group (aPR=1) was general population men.

[b]including adjustment for covariates in Model I plus other drug use in past 12 months.

*P <.05
